# African genetic ancestry interacts with body mass index to modify risk for uterine fibroids

**DOI:** 10.1371/journal.pgen.1006871

**Published:** 2017-07-17

**Authors:** Ayush Giri, Todd L. Edwards, Katherine E. Hartmann, Eric S. Torstenson, Melissa Wellons, Pamela J. Schreiner, Digna R. Velez Edwards

**Affiliations:** 1 Division of Epidemiology, Vanderbilt University Medical Center, Nashville, Tennessee, United States of America; 2 Institute for Medicine and Public Health, Vanderbilt University Medical Center, Nashville, Tennessee, United States of America; 3 Vanderbilt Genetics Institute, Vanderbilt University School of Medicine, Nashville, Tennessee, United States of America; 4 Department of Obstetrics and Gynecology, Vanderbilt University School of Medicine, Nashville, Tennessee, United States of America; 5 Vanderbilt Epidemiology Center, Vanderbilt University Medical Center, Nashville, Tennessee, United States of America; 6 Division of Diabetes, Endocrinology and Metabolism, Vanderbilt University Medical Center, Nashville, Tennessee, United States of America; 7 Division of Epidemiology and Community Health, University of Minnesota, Minneapolis, Minnesota, United States of America; Icahn School of Medicine at Mount Sinai, UNITED STATES

## Abstract

Race, specifically African ancestry, and obesity are important risk factors for uterine fibroids, and likely interact to provide the right conditions for fibroid growth. However, existing studies largely focus on the main-effects rather than their interaction. Here, we firstly provide evidence for interaction between categories of body mass index (BMI) and reported-race in relation to uterine fibroids. We then investigate whether the association between inferred local European ancestry and fibroid risk is modified by BMI in African American (AA) women in the Vanderbilt University Medical Center bio-repository (BioVU) (539 cases and 794 controls) and the Coronary Artery Risk Development in Young Adults study (CARDIA, 264 cases and 173 controls). We used multiple logistic regression to evaluate interactions between local European ancestry and BMI in relation to fibroid risk, then performed fixed effects meta-analysis. Statistical significance threshold for local-ancestry and BMI interactions was empirically estimated with 10,000 permutations (p-value = 1.18x10^-4^). Admixture mapping detected an association between European ancestry and fibroid risk which was modified by BMI (continuous-interaction p-value = 3.75x10^-5^) around *ADTRP* (chromosome 6p24); the strongest association was found in the obese category (ancestry odds ratio (AOR) = 0.51, p-value = 2.23x10^-5^). Evaluation of interaction between genotyped/imputed variants and BMI in this targeted region suggested race-specific interaction, present in AAs only; strongest evidence was found for insertion/deletion variant (6:11946435), again in the obese category (OR = 1.66, p-value = 1.72x10^-6^). We found nominal evidence for interaction between local ancestry and BMI at a previously reported region in chromosome 2q31-32, which includes *COL5A2*, and *TFPI*, an immediate downstream target of *ADTRP*. Interactions between BMI and SNPs (single nucleotide polymorphisms) found in this region in AA women were also detected in an independent European American population of 1,195 cases and 1,164 controls. Findings from our study provide an example of how modifiable and non-modifiable factors may interact to influence fibroid risk and suggest a biological role for BMI in fibroid etiology.

## Author summary

Although it is postulated that obesity and non-modifiable risk factors such as race or genetic ancestry may interact to jointly influence uterine fibroid growth, most existing studies have not evaluated their interaction. In this study we exhibit evidence for interaction across several tiers of investigation. We first show that the association between reported/third-party identified-race (African American (AA) and European American (EA)) and fibroid risk is modified by body mass index (BMI) categories. We then reveal evidence for interaction between two genetically-inferred local European ancestry regions (top two regions: chromosome 6p24, *ADTRP*; chromosome 2q31-32, *TFPI*, *COL5A2*) along the genome and BMI in relation to fibroids in two independent AA populations. Of intrigue, genes in these top two regions are mechanistically related, where *ADTRP* gene-product is an immediate upstream regulator for *TFPI*. Then at the genotype level, we show that interaction between genotyped/imputed variants and BMI is race-specific for chromosome 6p24 region, present in AAs but not in EAs, whereas, trans-ethnic, common across AAs and EAs for the 2q31-32 region, as replicated in an independent population of EA women. Our multi-tiered investigation supports evidence for interaction between reported race, genetic ancestry and BMI in relation to fibroid risk.

## Introduction

Uterine leiomyomata, also referred to as fibroids, are benign growths arising from myometrial smooth muscle cells. As the most common pelvic tumor in women, prevalence of fibroids ranges from 20 to 77% [1–5], and accounts for $9.4-$34 billion dollars annually in healthcare costs [5–7]. As the leading indication for hysterectomy (33%) in US women of reproductive age, it represents a significant source of burden for women and the healthcare system [8].

There are large disparities in fibroid risk across racial and ethnic populations [8]. Compared with European American (EA) women, African American (AA) women are two to three times more likely to be diagnosed with fibroids [2;3], which are also larger in size and greater in number [2;3;9]. AAs also have an approximately 10 year earlier onset of fibroids [4], are more likely to have hysterectomy and seven-fold more likely to have myomectomies for the treatment of fibroids [10]. The role of genetic predisposition in this disparity is supported by two admixture mapping studies of AAs which demonstrated that greater proportion of European ancestry was inversely associated with fibroids in AA women [11;12].

Obesity is associated with higher fibroid risk with most studies reporting a positive but non-linear relationship with categories of body mass index (BMI), an association that may be mediated by elevated bioavailable estrogen and/or testosterone associated with obesity [13–18]. The role of endogenous sex hormones in the etiology of fibroids is widely accepted, with factors related to higher cumulative exposure showing increased risk, such as greater age at menopause, after which fibroid risk decreases, and earlier age at menarche [19]. Interestingly, the Uterine Fibroid Study (UFS) showed positive associations between categories of BMI and fibroids, irrespective of fibroid number or size in black women, but not in white women [18].

Non-modifiable factors such as race/ethnicity and genetics, and modifiable risk factors such as obesity likely interact together to provide the right conditions for fibroid growth. However, most existing studies relating to fibroids have largely focused on these factors individually rather than their interaction. Recognizing this crucial gap in the literature, the primary goal of this study was to evaluate interactions between local genetic ancestry across the genome and BMI in relation to fibroid presence in AA women from the Vanderbilt University Medical Center (VUMC) Synthetic Derivative (SD) electronic medical record (EMR) database and bio-repository (BioVU), and the Coronary Artery Risk Development in Young Adults (CARDIA) study.

## Results

**Fig 1:**
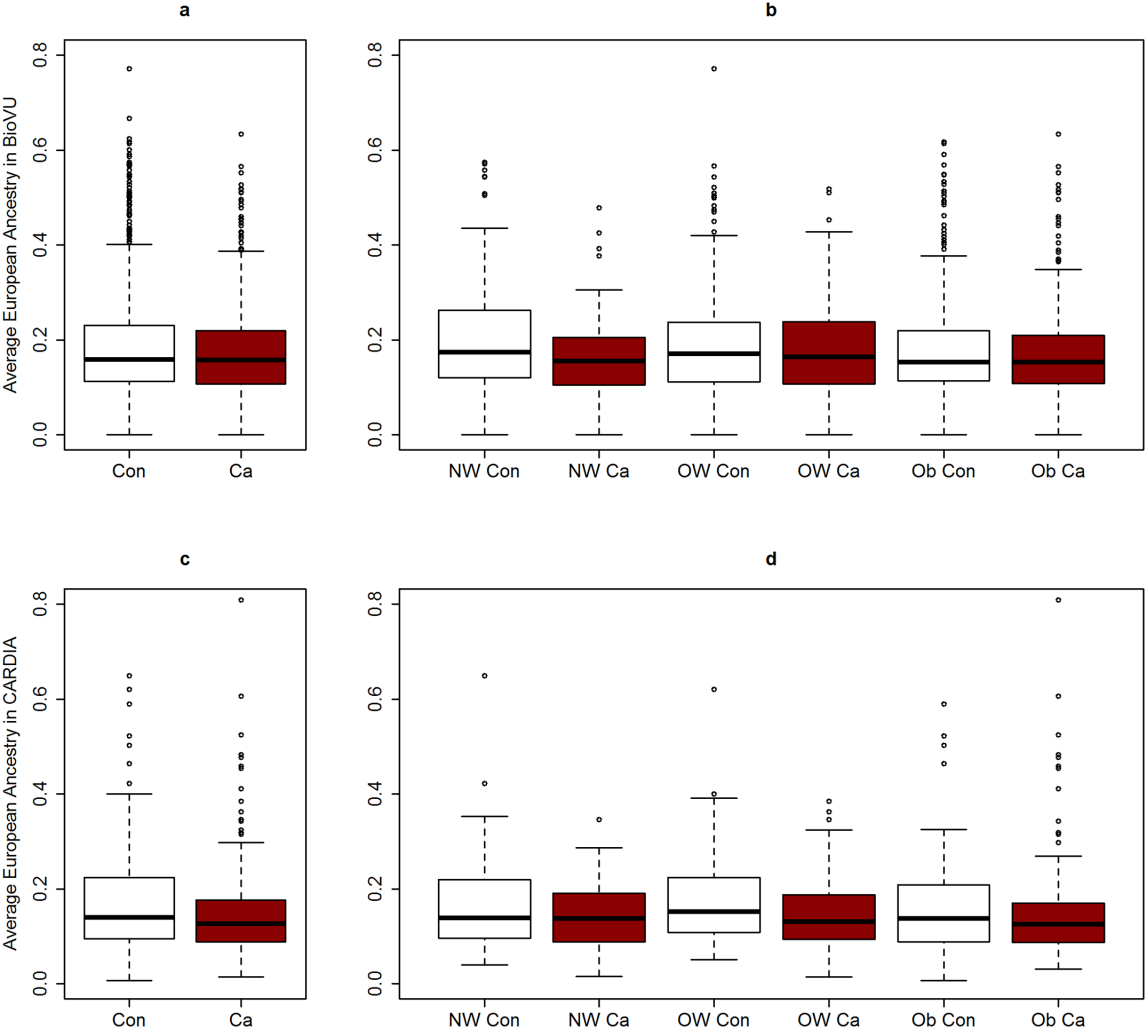
Box plot of average European ancestry estimates in women from BioVU and CARDIA. Con = Controls; Ca = Cases; NW = normal-weight; OW = over-weight; Ob = obese; Average European ancestry by case control status in (a) BioVU, and (b) CARDIA; Average European ancestry by case-control status across BMI categories in (c) BioVU and (d) CARDIA.

Comparing women with data available on fibroid status and genotype information in BioVU and CARDIA, cases were more likely to be obese (59.2% and 58.7% in BioVU and CARDIA, respectively) than controls (50.7% and 47.3% in BioVU and CARDIA, respectively) (Table 1). Average age at fibroid diagnosis was 40.6 in the BioVU and 40.0 in the CARDIA; age distribution of controls was similar to those of cases (Table 1). On average, cases had lower proportion of average European ancestry across the genome than controls (Table 1). Similar trends of lower European ancestry in cases versus controls were observed when median European ancestry was visualized by strata of BMI category (Fig 1a–1d). Distribution of characteristics of cases and controls in the larger SD and CARDIA datasets (including women with and without genotype data) were similar to those described above (Table 1). AA race was positively associated with fibroids in both the SD and CARDIA, as expected (Table 1). AA women in BioVU with genetic data available were comparable to AA women in the larger SD, with the exception of age, where average age was more balanced across cases and controls in BioVU than in the larger SD (S1 Table).

**Table 1:** Characteristics of fibroid cases and controls in the Vanderbilt University Synthetic Derivative (SD) and CARDIA.

**Participants**	**Synthetic Derivative**^a^		**P**	**CARDIA**^b^		**P**
	**All cases**	**All controls**		**All cases**	**All controls**	
Continuous	Mean (SD)	Mean (SD)		Mean (SD)	Mean (SD)	
Age	45.9 (11.5)	39.5 (16.1)	<0.0001	42.3 (3.6)	41.9 (3.8)	0.028
BMI	32.4 (9.7)	30.4 (8.7)	<0.0001	30.9 (7.6)	28.9 (8.2)	0.0001
Categorical	N (%)	N (%)		N (%)	N (%)	
Race			<0.001			<0.001
Black	1,309 (32.7)	2,573 (20.8)		398 (65.6)	147 (33.0)	
White	2,698 (67.3)	9,783 (79.2)		209 (34.4)	299 (67.0)	
BMI			<0.001			<0.001
<25kg/m2	846 (21.1)	3,728 (31.2)		144 (23.7)	182 (40.8)	
25–30 kg/m2	1,048 (26.2)	3,318 (26.8)		179 (29.5)	99 (22.2)	
30–35 kg/m2	867 (21.6)	2,360 (19.1)		128 (21.1)	80 (17.9)	
>35 kg/m2	1,246 (31.1)	2,950 (23.9)		156 (25.7)	85 (19.1)	
Participants	**BioVU**^c^			**CARDIA**^d^		
	**AA GWAS Cases**	**AA GWAS Controls**		**AA GWAS Cases**	**AA GWAS Controls**	
Continuous	Mean (SD)	Mean (SD)		Mean (SD)	Mean (SD)	
Age	40.6 (10.9)	41.7 (15.5)	0.144	40.0 (3.7)	39.0 (4.2)	0.763
BMI	33.4 (8.8)	31.8 (8.5)	0.001	32.9 (7.9)	31.4 (8.3)	0.380
Average European Ancestry (%)	17.5 (10.1)	19.2 (12.5)	0.008	14.8 (9.7)	17.5% (11.5)	0.112
Categorical	N (%)	N (%)	0.009	N (%)	N (%)	
BMI						0.054
<25kg/m2	83 (15.4)	141 (20.3)		33 (12.5)	36 (24.7)	
25–30 kg/m2	137 (25.4)	201 (29.0)		76 (28.8)	41 (28.1)	
>30 kg/m2	319 (59.2)	352 (50.7)		155 (58.7)	69 (47.3)	

^a^ Includes all eligible participants at Vanderbilt Synthetic Derivative (SD) with or without GWAS data;

^b^ Eligible CARDIA participants with or without GWAS data;

^c^ Subset of African Americans in the SD with available GWAS data;

^d^ Subset of African Americans in CARDIA with available GWAS data; AA = African Americans

In the larger SD and CARDIA datasets, we evaluated the association between reported race and fibroid risk stratified by BMI category (Table 2). In both datasets, EA women were less likely to have fibroids than AA women across all strata of BMI (Table 2). However, in the SD, this inverse association strengthened monotonically with increasing strata of BMI until the last strata. The magnitude of the inverse association between race and fibroid presence was smallest in the normal-weight (<25 kg/m^2^) strata (Odds Ratio (OR) = 0.53, 95% Confidence Interval [CI] 0.43, 0.64), and largest in the obese (30–35 kg/m^2^) strata (OR = 0.36, 95% CI 0.30, 0.43). Formal tests for interaction using the likelihood ratio test showed evidence for effect modification between categories of BMI and reported race (P = 0.01). Visual evaluation of the interaction in the SD by strata of race, with odds ratios representing odds of fibroids across categories of BMI showed a steeper slope for increased odds in African Americans than for European Americans, the source of interaction (S1 Fig and S2 Table). With far fewer cases and controls per strata compared to the SD, this trend was not similar in CARDIA. We then evaluated the association between genetically inferred global ancestry and uterine fibroids in the subset of African American women in the SD with genetic data available (BioVU) and in CARDIA. Every 10% increase European ancestry was associated with 0.88 (95% CI: 0.80, 0.97) and 0.86 (95% CI: 0.71, 1.03) decreased odds in uterine fibroids in BioVU and CARDIA, respectively (S3 Table). European ancestry was inversely associated with uterine fibroids across each strata of BMI category, however, there was no significant trend for interaction notable for global ancestry (S3 Table).

**Table 2:** Association between race/ethnicity and fibroid presence by categories of BMI in the Vanderbilt University SD and CARDIA.

	Synthetic Derivative					CARDIA					Meta-analysis	
BMI Categories	N Cases/ Controls	OR^a^	(95% CI)	P^b^	P-int^c^	N Cases/ Controls	OR^a^	(95% CI)	P^b^	P-int^c^	OR	(95% CI)
<25kg/m2	846/3,728	0.52	(0.43, 0.64)	<0.001	0.01	144/182	0.18	(0.10, 0.32)	<0.001	0.75	0.47	(0.39, 0.56)
25–30 kg/m2	1,048/3,318	0.47	(0.39, 0.56)	<0.001		179/99	0.25	(0.14, 0.43)	<0.001		0.44	(0.37, 0.53)
30–35 kg/m2	867/2,360	0.36	(0.30, 0.43)	<0.001		128/80	0.36	(0.18, 0.71)	0.003		0.36	(0.30, 0.43)
>35 kg/m2	1,244/2,949	0.50	(0.43, 0.58)	<0.001		156/85	0.35	(0.19, 0.66)	0.001		0.49	(0.42, 0.57)

^a^OR: whites (comparison group), blacks (reference group)

^b^P: p-value from z-score for individual categories

^c^P-int: p-value for global interaction using likelihood ratio test fitting a reduced and a full model.

Reduced model: logit(Fibroids) ~ BMI-category-0 (<25kg/m2: Reference) + BMI-category1 (25–30 kg/m2) + BMI-category2 (30–35 kg/m2) + BMI-category3 (>35 kg/m2) + race (African (0) or European (1)) + Age.

Full Model: logit(Fibroids) ~ BMI-category-0 (<25kg/m2: Reference) + BMI-category1 (25–30 kg/m2) + BMI-category2 (30–35 kg/m2) + BMI-category3 (>35 kg/m2) + race (African (0) or European (1)) + BMI-category1 x race + BMI-category2 x race + BMI-category3 x race + Age.

**Fig 2:**
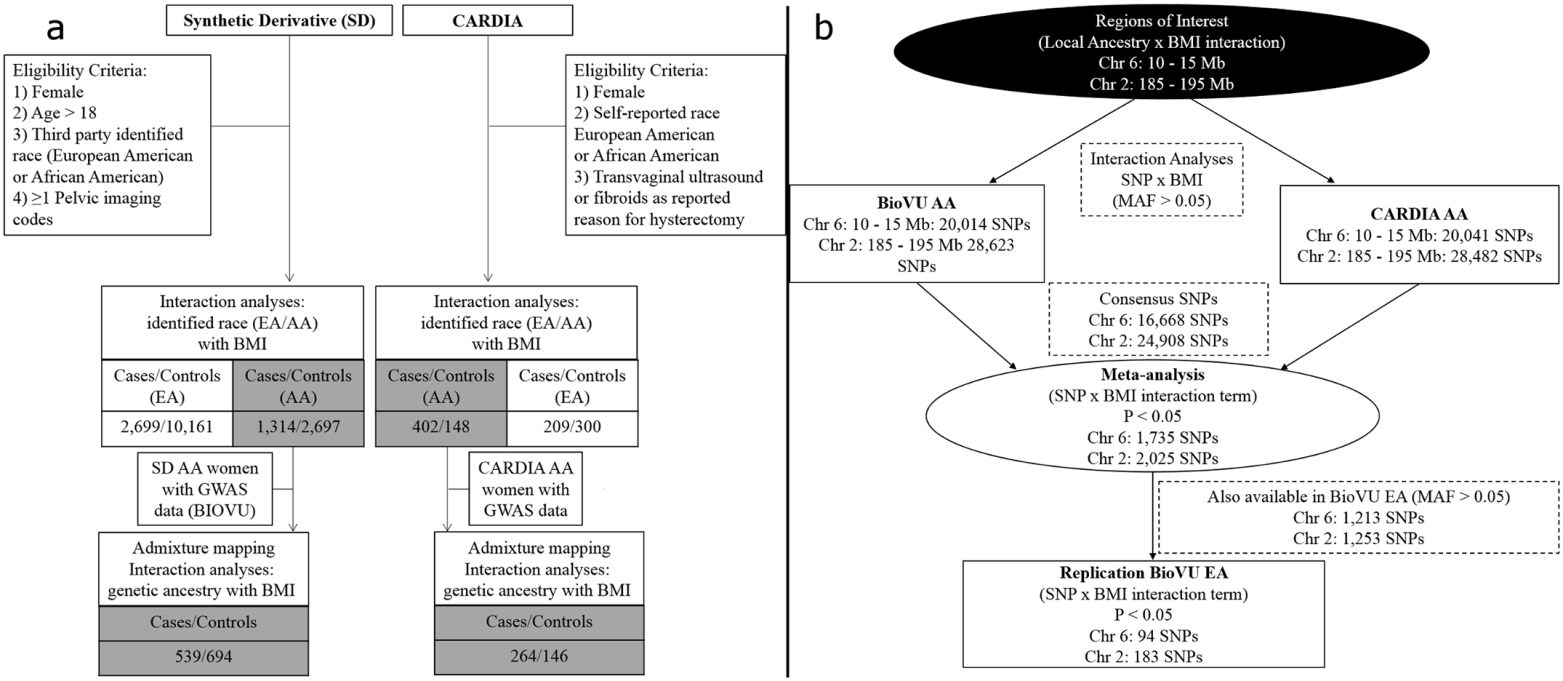
Flow chart of study sample and analyses. a) Flow chart focuses on AA populations used for global/local ancestry estimation and local-ancestry x BMI interaction analyses; b) Flow chart focuses on replication of BMI x SNP interactions in candidate regions using AA and EA populations.

**Fig 3:**
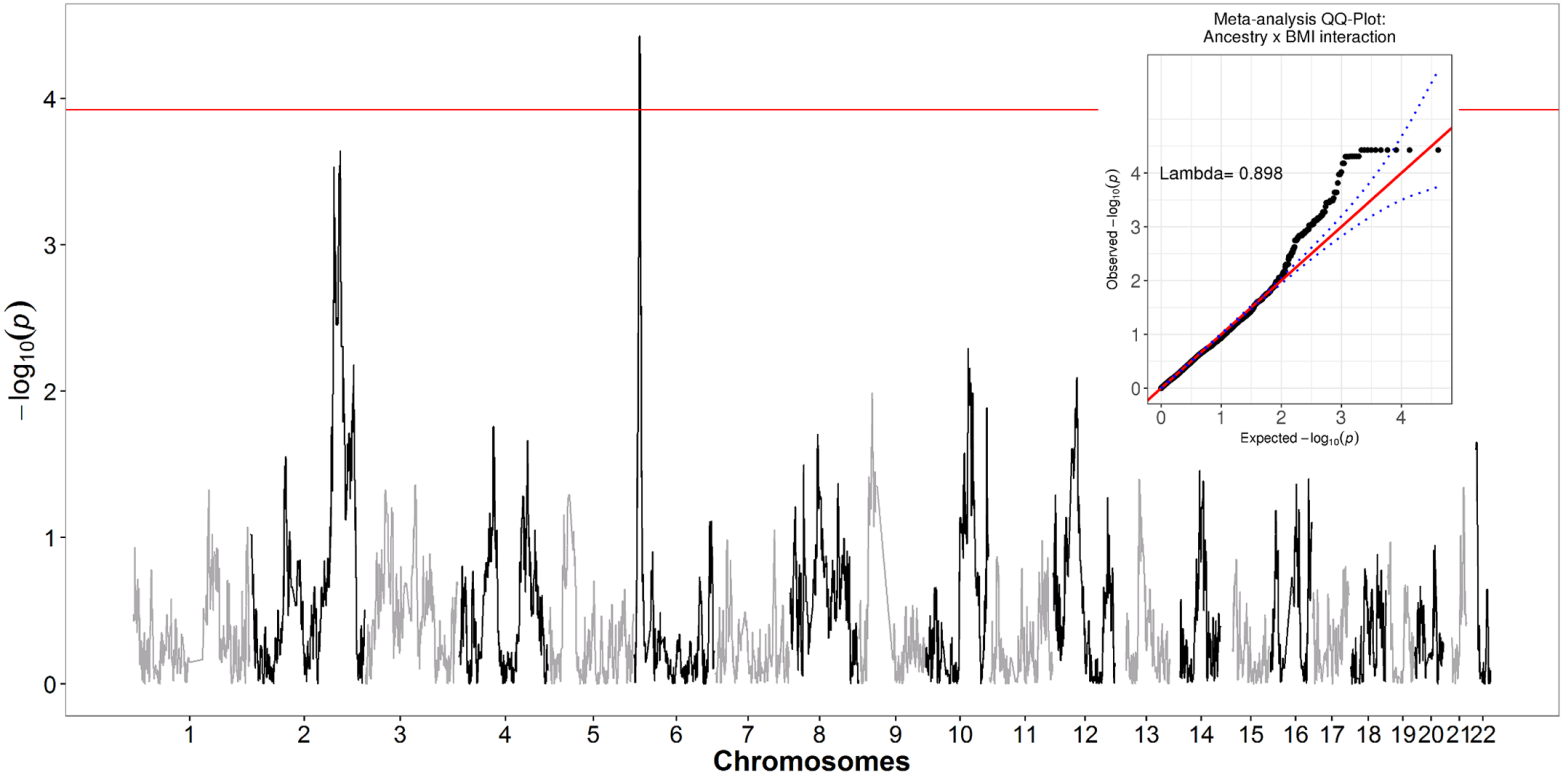
Meta-analysis of genome-wide local ancestry and BMI (continuous) interaction in African American women in BioVU and CARDIA. Negative log(10) p-values for fibroids modeled against local European ancestry and BMI with continuous interaction term (BMI x Local ancestry); y-axis reflects–log10(p) for the interaction term.

**Fig 4:**
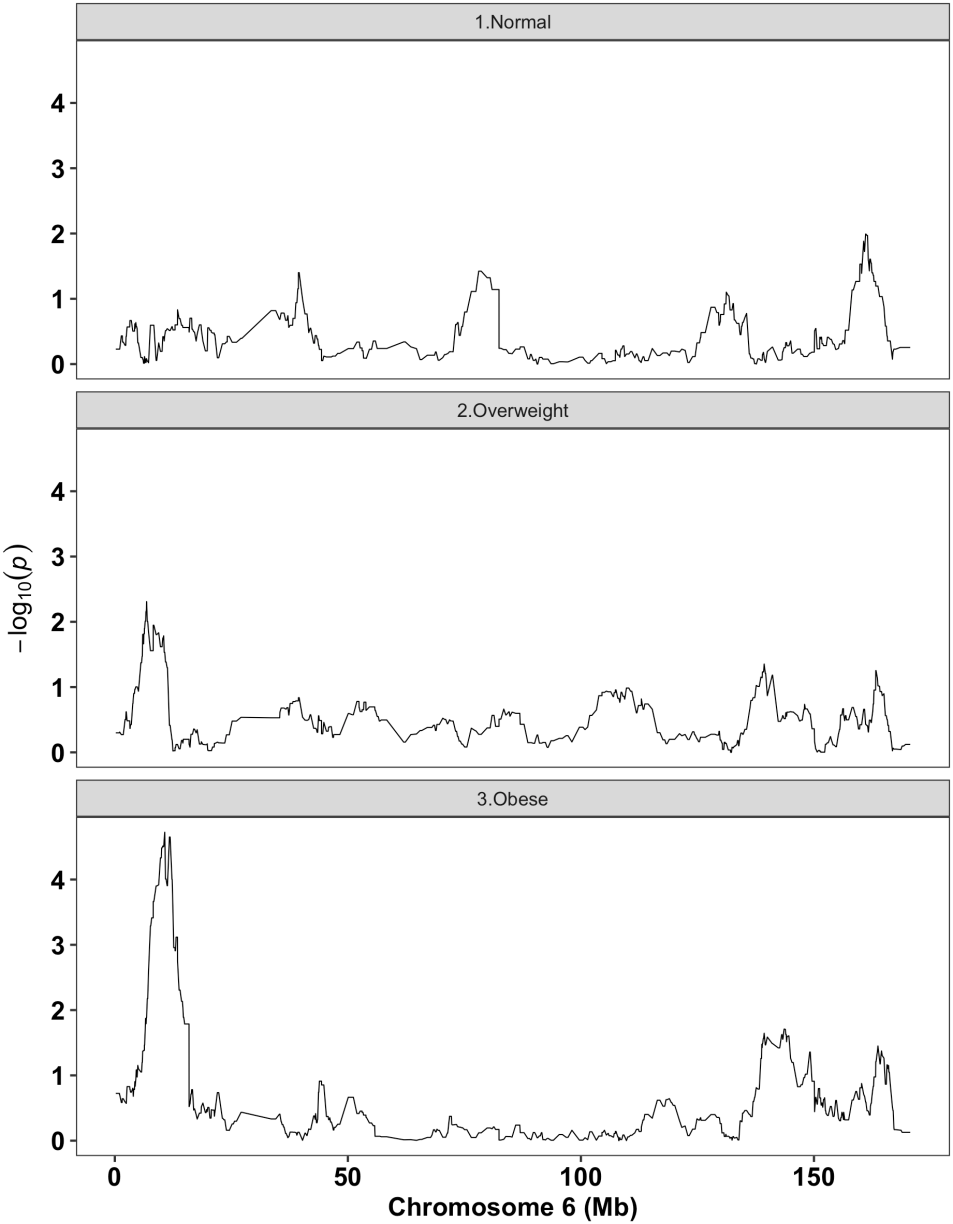
Local ancestry analyses in chromosome 6: Stratified by BMI categories. Negative log(10) p-values for fibroids modeled against local European ancestry by strata of BMI category.

We then evaluated interactions between BMI (continuous) and local European ancestry across the genome (inferred through genetic data) in relation to fibroids in BioVU AA and CARDIA AA women (Fig 2a) and then performed a fixed-effects meta-analysis (Fig 3). Local ancestry estimates in the chromosome 6p24 region showed strongest evidence of interaction with BMI in relation to fibroids (Fig 3 and S2 Fig). Although no interaction surpassed the canonical genome-wide significance threshold of 5x10^-8^, the strongest signal in the chromosome 6p24 region passed empirically estimated statistical significance threshold through 10,000 permutations (p-threshold = 1.18x10^-4^). Interaction ORs (BMI x local ancestry estimate) from meta-analysis at marker rs6457825 was 0.95 (P = 3.75x10^-5^; P for heterogeneity [Het-P] = 0.91) (Table 3). The association between local European ancestry at this marker and fibroids decreased monotonically with increasing categories of BMI (Table 3); the strongest admixture mapping signal was observed in the obese (BMI >30 kg/m^2^) category (Fig 4). Each unit increase in European ancestry at this marker was associated with 0.51 (P = 2.23x10^-5^; Het-P = 0.50) reduced odds of fibroids in the obese category (Table 3).

**Table 3:** Summary of empirically inferred local ancestry x BMI interaction analyses at top *ADTRP* SNP rs6457825 from 6p24: Continuous and stratified by BMI categories.

	BioVU			CARDIA			Meta					
	OR	95% CI	P	OR	95% CI	P	OR	SE	HetP	P	Direction	I^2^
*BMI x rs6457825	0.95	0.93–0.98	2.36x10^-4^	0.95	0.91–1.00	0.062	0.95	0.01	0.906	3.75x10^-5^	--	0%
BMI Category												
Overall	0.77	0.60–0.98	0.035	0.63	0.39–1.02	0.061	0.74	0.11	0.481	6.34x10^-3^	--	0%
<25kg/m^2^	1.39	0.74–2.61	0.302	1.15	0.26–5.04	0.85	1.35	0.29	0.815	0.306	++	0%
25–30 kg/m^2^	0.90	0.55–1.48	0.680	0.57	0.21–1.52	0.265	0.82	0.22	0.417	0.385	--	0%
>30 kg/m^2^	0.54	0.38–0.77	5.76x10^-4^	0.41	0.21–0.81	0.010	0.51	0.16	0.498	2.23x10^-5^	--	0%

SNP = single nucleotide polymorphism; OR = odds ratio; 95% CI = 95% confidence interval; Meta = Meta-analysis of two studies using inverse variance fixed effects model

BMIxrs6457825: refers to interaction odds ratio obtained from logistic regression model where logit(Fibroids) ~ local ancestry at rs6457825 (0, 1 or 2 European ancestry copies) + BMI (continuous) + BMI x local ancestry at rs6457825 + age + 10 principal components;

BMI category: refers to association between local ancestry at that marker and uterine fibroids without considering interaction with BMI across all individuals (overall), and among individuals with BMI <25 kg/m^2^, 25–30 kg/m^2^ or >30kg/m^2^

*Relevant estimates form the interaction model including those for BMI, local ancestry at rs6457825, and interaction of BMI x local ancestry at rs6457825 for BioVU and CARDIA are presented in S4 Table

**Fig 5:**
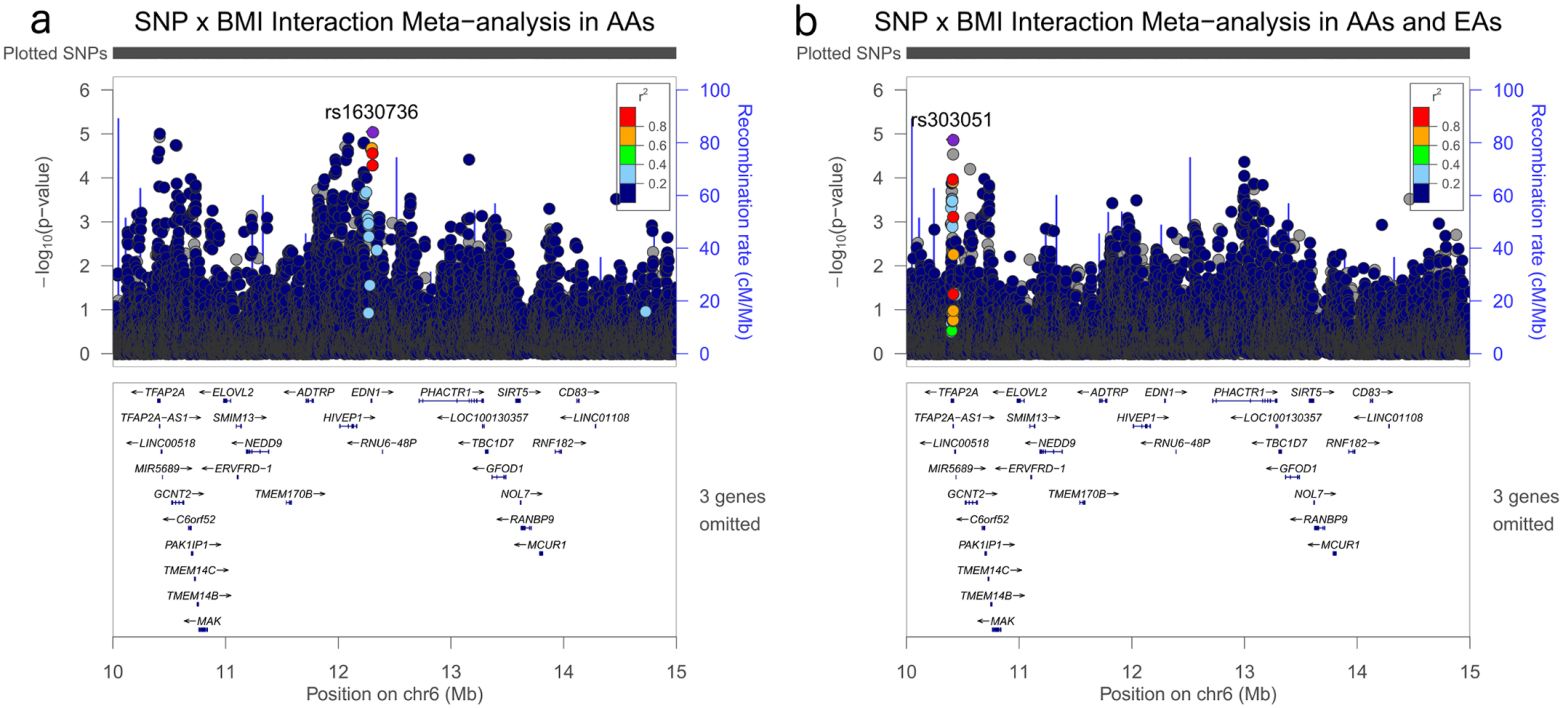
Regional association plots for SNP x BMI (continuous) interaction p-values for targeted region in chromosome 6 before and after meta-analysis with European American women. Plotted p-values are for BMI x SNP interaction term in the following scenarios: a) chromosome 6 region meta-analysis in BioVU AA and CARDIA AA; b) chromosome 6 region meta-analysis in BioVU AA and CARDIA AA + BIOVU EA.

Investigation of single nucleotide polymorphism (SNP) and BMI interactions (as a continuous term) using imputed/genotyped variants in the chromosome 6p24 region in AA specific datasets showed 1,735 SNPs had p-value less than 0.05 (Fig 1b), with the most-statistically significant SNPs hovering around the ADTRP gene (Fig 5a). Interaction analyses reflecting test-for-trend across categories of BMI, and stratified analyses suggested an insertion/deletion (indel) variant was associated with fibroid risk and the association differed by BMI strata (Table 4). Compared to the reference allele (CTT), each additive unit of the effect allele (C) for variant chr6:11946435, located in the Androgen Dependent TFPI Regulating Protein (*ADTRP*) gene was positively associated with fibroid presence in the obese category (Meta-analysis OR = 1.66; P = 1.72x10^-6^; Het-P = 0.83) (Table 4 and Supplemental S3 Fig). Consistent with the direction of association with local ancestry estimates in this region, the allele frequency for the effect allele (here, also the allele associated with greater odds for fibroids) is higher in the African populations (73%) than in the European populations (17%) from the 1000 Genomes reference panel. Conditioning this association on local ancestry at marker rs6457825 attenuated the OR in the obese strata from 1.66 to 1.46 (P = 7.70x10^-4^).

**Table 4:** Summary of strongest SNP/indel (6:11946435) association from BMI category and SNP interaction from 6p24.

	BIOVU AA			CARDIA AA			Meta-analysis AA			BIOVU EA (Replication)		
Interaction Type	OR	SE	P	OR	SE	P	OR	P	I^2^	OR	SE	P
BMI categories (0, 1, 2) x SNP^a^	1.49	0.11	4.92x10^-4^	1.55	0.22	5.07x10^-2^	**1.50**	**6.54x10**^**-5**^	**0**	1.02	0.10	8.34x10^-1^
BMI (continuous) x SNP	1.03	0.01	1.12x10^-2^	1.04	0.02	1.13x10^-1^	**1.03**	**2.99x10**^**-3**^	**0**	1.00	0.01	9.58x10^-1^
BMI (per SD) x SNP^b^	1.26	0.09	1.11x10^-2^	1.33	0.18	1.12x10^-1^	**1.27**	**3.00x10**^**-3**^	**0**	1.01	0.09	9.60x10^-1^
**BMI Category**												
< 25 kg/m^2^	0.67	0.22	7.40x10^-2^	-	-	-	**-**	**-**	**-**	0.96	0.14	7.82x10^-1^
25–30 kg/m^2^	1.03	0.17	8.56x10^-1^	1.11	0.33	7.59x10^-1^	**1.05**	**7.62x10**^**-1**^	**0**	0.98	0.17	9.22x10^-1^
> 30 kg/m^2^	1.65	0.12	3.77x10^-5^	1.73	0.23	1.50x10^-2^	**1.66**	**1.72x10**^**-6**^	**0**	1.01	0.15	9.35x10^-1^

Effect allele (EA)/Reference allele (RA): C/CTT; Effect allele frequency in AFR (1000G): 0.73; Effect allele frequency in EUR (1000G): 0.17

^a^. Interaction was modeled between BMI-categories (0 = < 25 kg/m^2^; 1 = 25–30 kg/m^2^; 2 = > 30 kg/m^2^) and SNP, OR for this term represents the change in odds ratio for SNP in relation to fibroids across each category of BMI.

^b^. Interaction was modeled between standardized BMI (centered and divided by standard deviation) x SNP, OR for this term represents the change in odds ratio for SNP in relation to fibroids across each standard deviation increase in BMI

BMI Category: refers to associations between genetic variant and uterine fibroids among individuals with BMI <25 kg/m^2^, 25–30 kg/m^2^ or >30kg/m^2^

Of the 1,735 BMI-SNP interactions with p-value less than 0.05 in the AA specific meta-analyses, 1,213 SNPs were available and eligible for analysis in an independent imaging-confirmed replication dataset of European American (EA) women (1,195 cases and 1,164 controls) from BioVU. Approximately 7.8% of these SNPs (94 SNPs) had interaction p-values less than 0.05 in the BioVU EA set (Fig 1b). Peaks present in AA-specific interaction analyses were attenuated when BioVU EA interaction estimates were meta-analyzed together with AA-specific estimates (Fig 5a and 5b), as the magnitude of interaction estimates were also attenuated in BioVU EA for the top hits in this region (Supplemental S5 Table).

The second strongest signal in the genetically inferred local ancestry by BMI (continuous) interaction analyses in AA women was found in the chromosome 2q31-32 region at marker rs12999125 (Meta-analysis OR = 1.04; P = 2.29x10^-4^; Het-P = 0.77) (Table 5, S4, S5 and S6 Figs). Further investigation of this signal in analyses stratified by BMI category showed that European ancestry was inversely associated with fibroid risk in the normal weight category (Meta-analysis OR = 0.55; P = 5.79x10^-2^; Het-P = 0.92) and that this association trended from inverse to positive across increasing categories of BMI (Table 5).

**Table 5:** Summary of empirically inferred local ancestry x BMI interaction analyses at top chromosome 2q31-32 SNP rs12999125: Continuous and stratified by BMI categories.

	BioVU			CARDIA			Meta					
	OR	95% CI	P	OR	95% CI	P	OR	SE	HetP	P	Direction	I^2^
*BMI x rs12999125	1.04	1.02–1.07	1.46x10^-3^	1.05	1.00–1.11	0.063	1.04	0.01	0.772	2.29x10^-4^	++	0%
BMI Category												
Overall	0.89	0.70–1.13	0.328	1.08	0.69–1.77	0.728	0.93	0.11	0.447	0.480	-+	0%
<25kg/m^2^	0.54	0.27–1.09	0.083	0.58	0.15–2.30	0.438	0.55	0.32	0.923	0.058	--	0%
25–30 kg/m^2^	0.60	0.37–0.97	0.037	1.81	0.68–4.86	0.238	0.74	0.22	0.048	0.176	-+	74%
>30 kg/m^2^	1.25	0.90–1.73	0.186	1.04	0.53–2.03	0.916	1.20	0.15	0.628	0.216	++	0%

OR = odds ratio; 95% CI = 95% confidence interval; Meta = Meta-analysis of two studies using inverse variance fixed effects model

BMI x rs12999125: refers to interaction odds ratio obtained from logistic regression model where logit(Fibroids) ~ local ancestry at rs12999125 (0, 1 or 2 European ancestry copies) + BMI (continuous) + BMI x local ancestry at rs12999125 + age + 10 principal components;

BMI category: refers to association between local ancestry at that marker and uterine fibroids without considering interaction with BMI across all individuals (overall), and among individuals with BMI <25 kg/m^2^, 25–30 kg/m^2^ or >30kg/m^2^

*Relevant estimates form the interaction model including those for BMI, local ancestry at rs6457825, and interaction of BMI x local ancestry at rs6457825 for BioVU and CARDIA are presented in S4 Table

**Fig 6:**
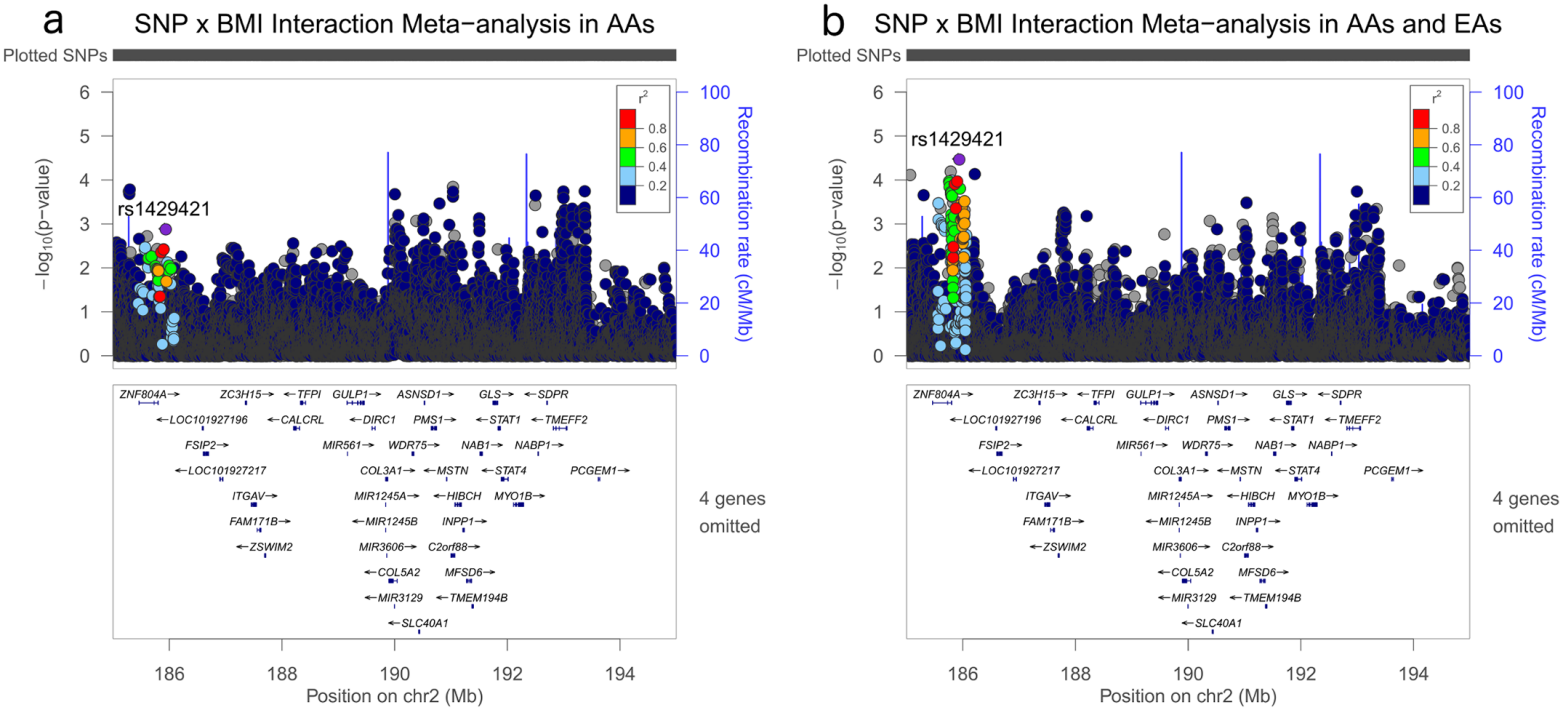
Regional association plots for SNP x BMI (continuous) interaction p-values for targeted region in chromosome 2 before and after meta-analysis with European American women. Plotted p-values are for BMI x SNP interaction term in the following scenarios: a) chromosome 2 region meta-analysis in BioVU AA and CARDIA AA; b) chromosome 2 region meta-analysis in BioVU AA and CARDIA AA + BIOVU EA.

Investigation of SNP and BMI interactions (as a continuous term) using imputed and genotyped variants in the chromosome 2q31-32 region in the AA specific datasets revealed 2,025 SNPs that had p-value less than 0.05, of which only 1,253 were available and eligible in the BioVU EA replication set. Approximately 15% of these (183 SNPs) also had interaction p-values less than 0.05 in the BioVU EA set (Fig 1b) and meta-analysis of AA and EA specific sets strengthened (Fig 6b and Supplemental S5 Table) the relatively weak signals observed in the AA-specific meta-analysis (Fig 6a). Interaction analyses as a continuous term, as a test-for-trend across categories of BMI, and stratified analyses showed SNP rs71430182 had the most consistent evidence across all three datasets as well as across all three methods of interaction assessed; strongest evidence was obtained with the BMI (continuous) x SNP interaction method (p = 7.15x10^-5^) (Table 6). Consistent with evidence in the local-ancestry-BMI interaction analyses, effect allele G was inversely associated with fibroids in the normal-weight (BMI < 25 kg/m2) category and the inverse association approached the null across increasing categories of BMI (Table 6). The effect allele is found in greater frequency in the European reference population (CEU EAF: 0.86) compared with their African counterpart (YRI EAF: 0.61). Conditioning the SNP-BMI interaction on local ancestry at marker rs71430182 for the AA specific analyses did not attenuate the effect estimate. For example, meta-analysis OR for AA specific analyses (BMI categories coded as 0, 1, 2 x SNP) decreased slightly from 1.37 to 1.36 when adjusted for rs71430182.

**Table 6:** Summary of strongest SNP (rs71430182) association from BMI category and SNP interactions from 2q31-32.

	BIOVU AA			CARDIA AA			BIOVU EA (Replication)			Meta-analysis AA + EA		
Interaction Type	OR	SE	P	OR	SE	P	OR	SE	P	OR	P	I^2^
BMI categories (0, 1, 2) x SNP ^a^	1.40	0.12	5.24x10^-3^	1.26	0.23	3.08x10^-1^	1.22	0.11	7.56x10^-2^	**1.29**	**7.75x10**^**-4**^	**0**
BMI (continuous) x SNP	1.02	0.01	3.44x10^-2^	1.04	0.02	4.98x10^-2^	1.04	0.01	3.74x10^-3^	**1.03**	**7.15x10**^**-5**^	**0**
BMI(per SD) x SNP ^b^	1.22	0.09	3.49x10^-2^	1.39	0.17	4.99x10^-2^	1.32	0.09	3.85x10^-3^	**1.28**	**5.80x10**^**-5**^	**0**
**BMI Category**												
< 25 kg/m2	0.73	0.17	6.43x10^-2^	-	-	-	0.74	0.16	5.19x10^-2^	**0.73**	**7.30x10**^**-3**^	**0**
25–30 kg/m2	0.83	0.15	2.02x10^-1^	0.89	0.30	6.91x10^-1^	0.97	0.18	8.60x10^-1^	**0.88**	**2.39x10**^**-1**^	**0**
> 30 kg/m2	1.13	0.10	2.42x10^-1^	0.99	0.21	9.58x10^-1^	1.08	0.16	6.40x10^-1^	**1.10**	**2.50x10**^**-1**^	**0**

Effect allele (EA)/Reference allele (RA): G/A; Effect allele frequency in AFR (1000G): 0.61; Effect allele frequency in EUR (1000G): 0.86

^a^. Interaction was modeled between BMI-categories (0 = < 25 kg/m^2^; 1 = 25–30 kg/m^2^; 2 = > 30 kg/m^2^) and SNP, OR for this term represents the change in odds ratio for SNP in relation to fibroids across each category of BMI.

^b^. Interaction was modeled between standardized BMI (centered and divided by standard deviation) x SNP, OR for this term represents the change in odds ratio for SNP in relation to fibroids across each standard deviation increase in BMI

BMI Category: refers to associations between genetic variant and uterine fibroids among individuals with BMI <25 kg/m^2^, 25–30 kg/m^2^ or >30kg/m^2^

## Discussion

The genetic basis of uterine fibroid risk and racial disparity in uterine fibroids are not well understood. Additionally, understanding of how modifiable risk factors such as obesity interact with non-modifiable risk factors such as race and genetic ancestry to influence uterine fibroid etiology is minimal. In this study, we first showed evidence of effect modification between BMI and reported race using the SD. Compared with AA women, EA women were less likely to have fibroids and effect sizes were stronger in the heavier BMI categories, with the strongest association found in the obese (BMI 30–35 kg/m^2^ category). Then using local ancestry estimates inferred from GWAS data in AA women, we show that BMI modifies the association between local European ancestry and fibroid risk in AA women from the BioVU and CARDIA study in two different genetic regions: chromosome 6p24 and chromosome 2q31-32. Further evaluation for evidence of interaction between SNPs around the chromosome 6p24 region and BMI suggested the signals were race-specific (only present in AA women, but not in EA women), which could be due to allelic heterogeneity and/or reduced detection power due to differences in allele frequency for SNPs in this region. However, in chromosome 2q31-32, although we detected suggestive evidence of interaction between BMI and local ancestry followed by suggestive evidence of interaction between BMI and SNPs in the region in AA women, incorporating EA women strengthened evidence for interaction between BMI and SNPs suggesting a common mechanism of action for this region across these two populations in relation to fibroids.

*ADTRP* is an androgen dependent gene [20] that regulates the expression of Tissue Factor Pathway Inhibitor (*TFPI*) [21;22], the gene product of which is a natural antagonist of Tissue Factor (TF). TF, a trans-membrane glycoprotein, is the main trigger of the blood coagulation cascade [23]. Non-membrane bound isoforms of TF have been shown to trigger angiogenesis and anti-apoptotic activity [24]. TF and TFPI proteins are found in the myometrial tissue [25]. With regard to obesity, TF concentration in the blood is higher in obese individuals, and has been shown to be reversible with weight reduction [26]. Additionally, considering androgen dependency of *ADTRP* mRNA expression [20], our finding of the strongest association in obese women is paralleled with the evidence that obese women have higher average circulating levels of testosterone compared with non-obese women [26]. Furthermore, our results extend etiological evidence to findings from a recent study that showed a positive association between higher free-testosterone levels and incident uterine fibroids, but an inverse association with recurrent fibroids [27]. The mechanistic relationships between obesity, androgens, *ADTRP*, *TF* and *TFPI* have not been studied in relation to uterine fibroids, and the relationships we detect here require additional functional studies.

The second BMI-local ancestry interaction that we noted in chromosome 2q31-q32 region, although only marginally significant, harbors several genes with relevance to fibroids including *COL3A1* and *COL5A2* as the closest genes (S5 Fig). One of the widely hypothesized models for fibroid formation has been related to abnormal tissue repair, disordered healing and altered extracellular matrix formation in parallel with keloid formation. Over expression of *COL3A1* mRNA [28–30], a key component in extracellular matrices, along with over-expression of *COL5A2* mRNA and higher presence of irregularly aligned collagen fibrils have been noted in fibroid-tissue compared with normal myometrial tissue [30]. Furthermore, both these genes have been found to be highly expressed in transformed fibroblasts in the Genotype-Tissue Expression (GTEx) Project database. Studies have shown abnormal over-expression of basic fibroblast growth factor (bFGF) mRNA [31] and bFGF ligand-receptor [32] in fibroid tissues compared with matched normal-myometrial tissue.

In addition to *COL3A1* and *COL5A2*, it is noteworthy that *TFPI* also lies within this admixture mapping peak and is within 1.75 Mb of the top chromosome 2 signal (S5 Fig). The observation that the strongest signal in this analysis (*ADTRP*) is a potent activator of the *TFPI* gene, which is the second strongest signal in the analysis further strengthens the hypothesis that obesity and testosterone related pathways may modify fibroid risk through these genes. Further adding evidence for *TPFI* as a causal candidate gene for uterine fibroids, a study comparing gene expression between uterine fibroid tissue and normal myometrial tissue reported 3.9 times lower expression of the *TFPI* gene in fibroid tissues, on average [33].

We took several measures at the design and analysis stages of this study to ensure internal and external validity of study findings. First, we designed a novel approach for gene discovery at various levels of ancestry variables to find evidence: reported-race, global-ancestry and local ancestry. We used two independent data sources to provide interval validation of study results, where possible. We implemented several steps to reduce misclassification of fibroid status, an important and difficult consideration for uterine fibroids research. We limited the choice of our data sources to individuals for who fibroid status was confirmed through imaging modalities. Almost all cases in CARDIA were confirmed though transvaginal ultrasound (TVUS) and a few through hysterectomy reports. Importantly, all controls were also confirmed for absence of fibroids through TVUS in CARDIA. An even more rigorous algorithm was applied for the SD/BioVU, where cases were confirmed to have uterine fibroids through imaging reports and controls were required to have two or more imaging reports free of uterine fibroids. To minimize possibility of reverse causality, we considered an average BMI measure for the SD/BioVU participants that was reflective of their adult life up until the time of fibroid diagnosis (BMI points during pregnancy were excluded). For CARDIA, a more traditional cohort design, we took BMI measures at the time of the ultrasound visit, or the preceding visit if absent.

Despite these strengths, a few considerations are worth reflecting on while interpreting study results. Our statistical models included, age, principal components, BMI, local ancestry and the interaction term between BMI and local ancestry. We were unable to adjust for other potential confounding factors, such as smoking, parity, age at menarche and oral contraceptive use that may have influenced estimates for the interaction term. However, we considered the implications of this potential limitation thoroughly while designing the study. Simulation studies have shown bias to be a major issue when there is interaction between confounder and gene of interest, but that the association between confounder and outcome would have to be large, as would the interaction between confounder and genetic factor [34]. With the exception of race, we are not aware of other risk factors that have extremely large effect estimates on uterine fibroids. However, due to our inability to adjust for additional effects, albeit likely small effects, we are not able to completely rule out residual confounding. With regard to comparability of cases and controls between the SD and BioVU (smaller subset of the SD), fibroid cases and controls tended to have similar distributions across the SD and BioVU with regard to BMI but not with regard to age. Fibroid controls in BioVU tended to be a slightly older subset of SD controls. Therefore, the BioVU subset is not fully representative of the larger SD. However, this does not disrupt the internal validity of the global and local ancestry results. Instead, by having a slightly older set of controls, potential misclassification of would-be cases as controls is further minimized in the BioVU subset.

We did not find a statistically significant interaction between global ancestry and BMI in either BioVU or CARDIA. It is interesting that, although not significant, the direction of interaction across increasing categories of BMI for global ancestry is opposite than that noted for reported-race in the SD and the top hit for local ancestry in BioVU and CARDIA. Global ancestry provides an average of local ancestry associations, and in light of local ancestry results where we report interactions going in two different directions, it is likely that the genetic architecture of uterine fibroids is complicated at the least. In comparing results from imputed regions at the top two loci, it is notable that adjustment for local ancestry attenuated the odds ratio at the chromosome 6p24 region, but negligibly for the chromosome 2q31-32 region. Intuitively, the correlations between local ancestry and genotypes are higher for the chromosome 6p24 region which also had the greater ancestral allele frequency difference (0.17, and 0.73, in EUR and AFR respectively) than for the top SNP in the chromosome 2q31-32*f* region (0.86, and 0.61 in EUR and AFR, respectively). Therefore, adjustment for local ancestry at the top SNP had the largest impact on chromosome 6p24 than in chromosome 2q31-32 region.

Several findings from our study are in agreement, at least in part, with previously published findings. The UFS study showed a positive association between BMI and fibroids in AA women, but not in EA women, suggesting effect modification, even though a formal test was not conducted [18]. We also present our effect modification results as evaluations between BMI and fibroids by strata of race (EA and AA) (shown in S2 Table for SD, S6 Table for CARDIA). Compared with the normal-weight category, uterine fibroid ORs for overweight (BMI 25–30 kg/m^2^) and obese (BMI 30–35 kg/m^2^) EA women were lower than for AA women (S2 Table, S1 Fig). However, contrary to the UFS, effect estimates for the highest BMI category (BMI >35 kg/m^2^) were similar in EA and AA women. The effect modification was only apparent in the SD, which was considerably larger than the CARDIA sample size (S6 Table). The lack of consistency between the SD and CARDIA may reflect the difference in the types of fibroid cases between the two sets. The SD likely has greater number and proportion of symptomatic fibroid cases seeking care than the CARDIA, where most cases were incidental discoveries through TVUS examinations. In addition to the vast difference in sample sizes between the two studies, the populations are selected in a different manner, where one is based on EMRs, while the other is based on self-selection, people willing to participate in a cohort study. Furthermore, differences in cultural attitudes towards care seeking behaviors, especially for sensitive topics such as uterine fibroids may have further influenced effect estimates when comparing interaction between reported-race across the SD and CARDIA. It is intriguing though that interaction estimates across BioVU and CARDIA for genetically inferred ancestry, whether global or local ancestry, are similar across the two studies. In the same vein of thought, this is likely reflective of using a more objective approach to estimate ancestry within a group of women (African American women, regardless of the source population BioVU or CARDIA), instead of comparing women between reported-races.

To our knowledge, this is the first admixture mapping study to directly evaluate effect modification of the association between genetic ancestry and fibroids by BMI. Two additional admixture mapping studies have been previously conducted for uterine fibroids in AA women [11;12]. Similar to the two previous reports from the Black Women’s Health Study (BWHS) and the UFS, mean differences in average genetic European ancestry between cases and controls in BioVU and CARDIA ranged from 1.5% to 2.7% excess European ancestry in controls than in fibroid cases. Using BWHS, Wise and colleagues reported suggestive and statistically significant peaks in chromosomes 2, 4 and 10, with varying directions of risk associated with higher local European ancestry proportion [11]. Using the UFS, Zhang and colleagues reported suggestive associations between local ancestry and fibroids in chromosome 1q42.2 and 2q32.2, with one locus suggesting a positive association and the other suggesting a negative association between local European ancestry and fibroids [12]. As the third admixture mapping study relating to fibroids, our study corroborates suggestive evidence presented by the previous two studies for the chromosome 2q32.2 region and highlights the importance of further investigating this region. The nearest markers reported for this region are within 1Mb in proximity (rs256552 reported by Zhang et al.) and within 2.5 Mb (rs6710083 reported by Wise et al.) of the signal observed in this study (S6 Fig). Even though both these studies report excess European ancestry in uterine fibroid cases in chromosome 2q32 compared with the genome-wide average in cases, which is not in complete agreement with our observation, this is the only replicated region identified thus far with regards to admixture mapping. More notably, Zhang et al. show an increasing Z-score trend across increasing categories of BMI, where the effect of local European ancestry would again increase fibroid risk. Although the reason for discrepancy in the direction of association between this study and others is not completely clear, differences in study designs may provide a reasonable explanation. Previous admixture mapping studies used case-only approaches, which compute Z-scores at local ancestry in relation to global average, whereas we used a case-control design that allowed us to adjust for potential confounders in statistical models. Additionally, the primary focus of our study was different from previous studies; we focused on the interaction between BMI and local ancestry in relation to fibroids, while previous studies focused on association between local ancestry and fibroids. Availability of comparable results from previously published studies, evaluating local ancestry and BMI interaction in a case-control setting adjusted for global ancestry may facilitate future comparisons and may provide more insight into observed differences.

As the first admixture mapping study that formally evaluated interactions between local ancestry and BMI in relation to fibroid risk, we provide statistically significant evidence for interaction in the *ADTRP* gene and suggestive evidence for its immediate down-stream target, *TFPI*, in two independent samples of AA women from the BioVU and the CARDIA study. Our study further highlights the power and flexibility of admixture mapping to identify risk loci for uterine fibroids that are modified by obesity among AA women, a population that is likely at highest risk for fibroids. Further confirmation of these findings and further characterization of the mechanisms involved may suggest therapeutic approaches for this high risk population.

## Methods

### Study populations

Participants for this investigation were derived from the SD EMR database, located at VUMC, Nashville, TN, and from the CARDIA study. The Institutional Review Board at VUMC approved this study. Both the SD database and CARDIA study have been described in detail in previous publications [35;36]. Briefly, the SD consists of de-identified clinical data obtained from patients attending all clinics associated with the Vanderbilt University Medical Center hospital system. Clinical data are abstracted from multiple sources including diagnostic and procedure codes, basic demographics, discharge summaries, progress notes, health history, multi-disciplinary assessments, laboratory values, imaging reports, medication orders, and pathology reports. Women of age 18 years or older, with at least one diagnostic or procedural code for pelvic imaging in the SD, were considered to be eligible for case-control selection in this investigation [37].

CARDIA is a prospective cohort study that recruited 5,115 EA and AA participants (54.5% female) between 18–30 years of age at baseline from the years 1985–86 in four clinical centers (Birmingham, AL; Chicago IL; Minneapolis, MN; and Oakland, CA) in the US. Participant characteristics were collected and various health outcomes were measured at baseline and during follow-up visits at years 2, 5, 10, 15, 20, 25 and 30 [35]. Additionally, at year 16, the CARDIA Women’s Study (CWS), performed ancillary to the existing study, administered TVUS examinations to non-pregnant women who had attended the year 15 exam and had at least one intact ovary by self-report. Women with TVUS examination at year 16 or with additional information on fibroid presence in previous visits were the target population of this investigation. Analyses were first conducted using the SD, then replicated using CARDIA samples, followed by a meta-analysis where applicable.

### Case-control definitions

Cases and controls in the SD were selected using an algorithm described in detail previously [37]. Women who are at least 18 years of age with EMR data in the SD with at least one procedural code for imaging with ultrasound, magnetic resonance imaging, or computed tomography were eligible for selection. Women with at least one diagnostic/procedure code for fibroids, defined by the International Classification of Diseases 9 (ICD-9) or by the Current Procedural Terminology (CPT) criteria, were considered cases. To be considered as controls, women needed to have pelvic imaging codes in at least two different time points with no ICD-9, or CPT code indicative of fibroid presence, and no mention of fibroids related key words or hysterectomy related keywords in imaging reports, operative reports, pathology summaries, or in the Problem List in patient file. We identified 1,314 AA cases, 2,697 AA controls, 2,699 EA cases and 10,161 EA controls in the SD with or without genotyping data available.

In the CWS, for women who received a TVUS examination, trained staff recorded information on the number of fibroids, and largest fibroid dimension from three perpendicular planes. For this investigation, women with one or more fibroids of any size were considered cases and women with no fibroids as controls. Additionally, several women who were eligible for CWS but had indicated fibroids as a reason for hysterectomy from baseline till year 15 were considered cases. In CWS, there were 402 AA cases (88 self-reported hysterectomy), 148 AA controls, 209 EA cases (15 self-reported hysterectomy) and 300 EA controls, with or without genotyping data available.

### Covariates

For participants in the SD, age at first diagnosis of fibroids or first pelvic imaging with fibroids was noted for cases and age at last pelvic imaging without mention of fibroids was noted for controls. BMI was computed as the average BMI starting at age 18 and up until the time of fibroid diagnosis for cases and up until the last pelvic imaging without fibroids for controls. BMI during pregnancy was excluded from average calculations. The BMI variable computed from the SD thus reflects the mean non-pregnant BMI in cases and controls over time. Race was coded as AA or EA based on third-party identification in the SD EMR.

For women who were part of the CWS, age was recorded and BMI was directly measured for cases and controls during the study visit at year 16. For the small proportion of cases that were identified by self-reported reason for hysterectomy, age and BMI were determined using CARDIA visits that were closest to the time of hysterectomy. Race was recorded as AA or EA based on self-report at baseline in CARDIA and validated at the Year 2 examination.

### Genotyping and quality control (QC)

Fibroid cases and controls identified as AA or EA in the SD with DNA available and proper consent to use DNA for research (BioVU) were genotyped in the Affymetrix Axiom Biobank Genotyping Array and the Affymetrix Axiom World Array 3 (Affymetrix Inc., Santa Clara, CA, USA). CARDIA cases and controls were genotyped with the Affymetrix Genome-Wide Human single nucleotide polymorphism (SNP) Array 6.0 (Affymetrix Inc., Santa Clara, CA, USA). Standard QC measures were taken for genotyping data from AA women for both datasets using PLINK [38]. Individuals with low genotyping rates (<95%) were removed from consideration followed by SNPs with low genotyping quality (<95%). Individuals with inconsistency in reported versus genetically determined sex were excluded, followed by individuals with first degree or higher relatedness identified by identity-by-descent sharing from a random selection of approximately 100,000 autosomal SNPs. SNPs with minor allele frequencies less than 1% and SNPs that deviated from the Hardy-Weinberg equilibrium at p <10^−6^ threshold (in controls) were excluded. Upon QC completion, limiting to individuals with complete information on key covariates, there were 1,233 AA women (539 cases and 694 controls) and 410 AA women (264 cases and 146 controls) available for primary analysis in the BioVU and CARDIA, respectively. Additionally, data were available from 2,359 EA women from the BioVU (1,195 cases and 1,164 controls) after QC which were used for replication of interaction estimates for select markers from candidate regions, described below in the Statistical analysis section.

### Global and local ancestry estimation

We first identified consensus autosomal SNPs present in both the BioVU and CARDIA post-QC datasets. For consensus SNPs, we further limited inclusion to SNPs with allele-frequency differences (delta) >0.2 between the 1000 Genomes African and European populations. A total of 20,000 SNPs were identified, which were then used for local and global ancestry estimation.

Local ancestry estimation was performed using Local Ancestry in admixed Populations Ancestry (LAMP-ANC) with proxy ancestral allele frequency inputs for SNPs from Europeans and Africans to infer local ancestry across the genome [39]. We used allele frequency estimates from the African and European populations nested in the Phase 3 1000 Genomes reference panels as ancestral allele frequencies for Africans and Europeans for the 20,000 consensus markers described above [40]. The following settings and parameters were assumed: LD-pruning was set to r-squared value of 0.1, recombination rate was set to 1x10^-8^, time since admixture was assumed to be seven generations and the proportion of admixture estimates was set to 0.2 for European ancestry. For the resulting output from LAMP-ANC, local ancestry was then coded as 0, 1 or 2 European ancestry calls for each marker. Global ancestry (average European ancestry per individual) was then calculated by summing the local ancestry calls across the genome and dividing by the total number of markers used in ancestry estimation. Local and global ancestry estimates were inferred separately for BioVU and CARDIA. Comparison of the top principal component from EigenSoft [41] with global ancestry estimates showed strong correlations, 98% in BioVU and 99% in CARDIA (S7a & S7b Fig).

### Statistical analyses

We used multiple logistic regression to evaluate the association between reported race and fibroid risk in EA and AA women by strata of BMI categories (BMI < 25 kg/m^2^, 25–30 kg/m^2^, 30–35 kg/m^2^, and > 35 kg/m^2^) in the SD and CARDIA datasets, separately. Interaction between BMI and race was evaluated by performing the likelihood ratio test in StataIC, version 12 (StataCorp, College Station, TX, USA), obtained by comparing the following reduced and full models. In the reduced model uterine fibroids was modeled against race (0 for African American and 1 for European), and k-1 BMI category indicator variables (BMI 25–30 kg/m^2^, 30–35 kg/m^2^, and > 35 kg/m^2^, with BMI <25/m^2^ serving as the reference variable), adjusted for age. The full model included variables in the reduced model and also included interaction terms between race (0, and 1) and the three BMI indicator categories to provide 3-degrees of freedom for the likelihood ratio test. Estimates for the association between race and uterine fibroids for individuals in each BMI category was obtained from the full model. Equivalently, the association between categories and BMI and uterine fibroids for individuals in each race category were also obtained from the full model. A flow chart detailing primary study populations by analysis type and availability of GWAS data is shown in Fig 2a. To evaluate the potential impact of including cases based on self-reported hysterectomy due to fibroids with TVUS confirmed cases, we performed sensitivity analyses by excluding TVUS non-confirmed cases. As interaction estimates and trends were similar for both types of analyses (S7 Table) we opted to include fibroid cases ascertained by hysterectomy status in the remaining analyses.

For primary analyses, in the smaller subset of BioVU AA and CARDIA AA women for whom local ancestry estimates were inferred, we performed interaction analyses between BMI (continuous) and local ancestry estimates (additive model: 0, 1 or 2 copies of European ancestry) across the genome in a multiple logistic regression framework while adjusting for age, and first 10 principal components (Eigensoft), using PLINK software [38]. All analyses were performed separately for each of the two datasets. Resulting effect estimates were then aggregated using inverse variance-weighted fixed effects meta-analysis in METAL [42]. Threshold for statistical significance for admixture mapping interaction analyses was estimated using 10,000 min-p permutation tests. Briefly, case-control status was randomly shuffled in each iteration to break the association between markers/ancestry loci and outcome of interest in BioVU and CARDIA separately. Additionally, case-control status and the first principal component were paired and shuffled together in order to maintain the correlation between global ancestry and case-control status. Beta and standard errors resulting from each iteration were then aggregated using fixed-effects meta-analysis. The smallest interaction p-value across all markers from the meta-analysis in each iteration was stored and repeated for 10,000 iterations to yield 10,000 min-p test statistics. The p-value at the 5^th^ percentile of the rank-ordered statistics across the 10,000 test statistics constitutes the empirically estimated threshold for statistical significance (P = 1.18x10^-4^). Statistically significant and suggestive (P = 5x10^-4^) signals were further evaluated for the association between local European ancestry and fibroid presence by strata of BMI category (BMI < 25 kg/m^2^, 25–30 kg/m^2^, and >30 kg/m^2^) while adjusting for age and first 10 principal components.

Then to search for interactions between BMI and SNPs in relation to fibroids, we tested for interactions in the candidate regions using genotyped and imputed SNPs in the BioVU AA and CARDIA AA datasets (Fig 2b). We imputed 5–10 mega-base regions around the BMI-and-ancestry interaction peaks, using the Phase 3 1000 Genomes cosmopolitan reference panel using IMPUTE2 [43]. Genotyped and imputed variants with minor allele frequencies greater than 5% in these regions were then allowed to interact with BMI in the following ways: 1) BMI (continuous) x SNP for maximal detection power with Probabel [44], 2) BMI category (0, 1, or 2, as a continuous term) x SNP as a test for trend of association for SNP across meaningful BMI categories with Probabel, 3) standardized BMI (per standard deviation) x SNP,and 4) by strata of BMI for ease of interpretation with SNPTESTv2 [45]. Dataset specific analyses were then meta-analyzed using METAL. We estimated p-value threshold (p = 6.9x10^-6^) here by first estimating the effective number of independent tests with simpleM in each dataset (7,215 and 6,885 for BIOVU and CARDIA, respectively), and then dividing 0.05 by the estimate that was the more conservative of the two. For BMI-stratified analyses, where appropriate, conditional analysis adjusting for local ancestry marker with the strongest signal was conducted to evaluate whether the stratum specific association between SNP and fibroids was independent of local ancestry.

Finally, the subset of markers that interacted with BMI (continuous term) at a p-value threshold less than 0.05 in meta-analysis of AA specific datasets were tested for interaction in an independent dataset of European American women (BioVU EA) who were confirmed as fibroid cases (N = 1,195) and controls (N = 1,164) with pelvic imaging codes. Same tests of interaction were performed as with the AA specific datasets, although BMI (continuous) x SNP interaction was used as the primary test for statistical evidence for replication (p < 0.05) in BioVU EA.

## Supporting information

S1 Table Comparison of key characteristics for African American women in the SD and BioVU. (DOCX)

S2 Table Association between BMI and fibroid presence by race/ethnicity status in the Vanderbilt University Synthetic Derivative. (DOCX)

S3 Table Association between average European ancestry and uterine fibroids in African Americans from BioVU and CARDIA. (DOCX)

S4 Table Detailed estimates from top two regions from local ancestry x BMI (continuous) interaction models represented in Tables 3 and 5. (DOCX)

S5 Table Top 10 SNPs from meta-analysis of BMI x SNP interaction estimates from two candidate genetic-regions in BioVU AA, CARDIA AA and BioVU EA. (DOCX)

S6 Table Association between BMI and fibroid presence by race/ethnicity status in CARDIA. (DOCX)

S7 Table Sensitivity analyses comparing race and BMI interactions for CARDIA: Using CWS ultrasound confirmed cases and controls only vs. using ultrasound confirmed cases and controls and self-reported hysterectomy due to fibroids as additional cases. (DOCX)

S1 Fig Visual representation of interaction between reported race and BMI-categories in the Synthetic Derivative. (PDF)

S2 Fig Strongest ancestry by BMI interactions in meta-analysis: Chromosome 6. Negative log(10) p-values for fibroids modeled against local European ancestry and BMI with continuous interaction term (BMI x Local ancestry).
(PDF)

S3 Fig Regional association plots for SNPs under admixture mapping peak with the strongest overall and stratified by BMI categories. (PDF)

S4 Fig Association plot for local ancestry x BMI (continuous) interaction in chromosome 2. (PDF)

S5 Fig Regional association plot for local ancestry x BMI (continuous) interaction in chromosome 2q31-q32. (PDF)

S6 Fig Comparing proximity of markers from previously published admixture mapping signals for chromosome 2q31-q33. (PDF)

S7 Fig Comparison of average European ancestry estimates from LAMP local ancestry calls with the first principal component in African American women from a) BioVU and b) CARDIA. (PDF)
