# Supplementary material for: African genetic ancestry interacts with body mass index to modify risk for uterine fibroids
Source: PLoS Genet. 2017 Jul 17;13(7):e1006871. doi: 10.1371/journal.pgen.1006871 (PMC5536439; doi:10.1371/journal.pgen.1006871)
Supplement: S1 Table — (DOCX) [file pgen.1006871.s001.docx]

**Table S1: Comparison of key characteristics for African American women in the SD and BioVU.**

| Participants | **SD** | |  |  | **BioVU^c^** | |  |
| --- | --- | --- | --- | --- | --- | --- | --- |
|  | **All AA cases** | **All AA Controls** | **P** |  | **AA GWAS cases** | **AA GWAS Controls** | **P** |
| Continuous | Mean (SD) | Mean (SD) |  |  | Mean (SD) | Mean (SD) |  |
| Age | 42.6 (10.3) | 34.5 (15.2) | <0.0001 |  | 40.6 (10.9) | 41.7 (15.5) | 0.144 |
| BMI | 35.0 (10.0) | 33.6 (10.4) | 0.0001 |  | 33.4 (8.8) | 31.8 (8.5) | 0.001 |
| Average European Ancestry (%) | NA | NA |  |  | 17.5 (10.1) | 19.2 (12.5) |  |
|  |  |  |  |  |  |  |  |
| Categorical | N (%) | N (%) |  |  | N (%) | N (%) |  |
| BMI |  |  | <0.001 |  |  |  | 0.009 |
| <25kg/m2 | 160 (12.2) | 502 (19.5) |  |  | 83 (15.4) | 141 (20.3) |  |
| 25-30 kg/m2 | 271 (20.7) | 571 (22.2) |  |  | 137 (25.4) | 201 (29.0) |  |
| >30 kg/m2 | 878 (67.1) | 1,500 (58.3) |  |  | 319 (59.2) | 352 (50.7) |  |
