# Supplementary material for: African genetic ancestry interacts with body mass index to modify risk for uterine fibroids
Source: PLoS Genet. 2017 Jul 17;13(7):e1006871. doi: 10.1371/journal.pgen.1006871 (PMC5536439; doi:10.1371/journal.pgen.1006871)
Supplement: S2 Table — (DOCX) [file pgen.1006871.s002.docx]

**S2 Table: Association between BMI and fibroid presence by race/ethnicity status in the Vanderbilt University Synthetic Derivative**

| Variable | Whites (Total N = 14585) | | |  | Blacks (Total N = 4578) | | | P-int** |
| --- | --- | --- | --- | --- | --- | --- | --- | --- |
|  | OR | 95% CI | P |  | OR | 95% CI | P* |  |
| **BMI** |  |  |  |  |  |  |  | 0.0118 |
| <25kg/m2 | 1.00 | (Ref) |  |  | 1.00 | (Ref) |  |  |
| 25-30 kg/m2 | 1.34 | (1.19, 1.51) | <0.001 |  | 1.45 | (1.14, 1.84) | 0.002 |  |
| 30-35 kg/m2 | 1.41 | (1.24, 1.61) | <0.001 |  | 1.93 | (1.53, 2.44) | <0.001 |  |
| >35 kg/m2 | 1.93 | (1.71, 2.19) | <0.001 |  | 1.85 | (1.48, 2.30) | <0.001 |  |
| *P: P-value from Wald-test for individual categories | | | | | |  |  |  |
| **P-int: P-value for global interaction using likelihood ratio test | | | | | | |  |  |
