# Supplementary material for: African genetic ancestry interacts with body mass index to modify risk for uterine fibroids
Source: PLoS Genet. 2017 Jul 17;13(7):e1006871. doi: 10.1371/journal.pgen.1006871 (PMC5536439; doi:10.1371/journal.pgen.1006871)
Supplement: S3 Table — (DOCX) [file pgen.1006871.s003.docx]

**S3 Table: Association between average European ancestry and uterine fibroids in African Americans from BioVU and CARDIA**

|  | BioVU | | | |  | CARDIA | | | |
| --- | --- | --- | --- | --- | --- | --- | --- | --- | --- |
| BMI category | N Cases/Controls | OR* | (95% CI) | P |  | N Cases/Controls | OR* | (95% CI) | P |
| Overall | 578/804 | 0.88 | (0.80, 0.97) | 0.01 |  | 303/149 | 0.86 | (0.71, 1.03) | 0.108 |
| <25kg/m2 | 83/141 | 0.71 | (0.54, 0.93) | 0.01 |  | 56/24 | 0.61 | (0.37, 1.00) | 0.047 |
| 25-30 kg/m2 | 137/201 | 0.92 | (0.76, 1.10) | 0.362 |  | 93/32 | 0.87 | (0.58, 1.32) | 0.527 |
| >30 kg/m2 | 319/352 | 0.91 | (0.79, 1.04) | 0.18 |  | 154/93 | 0.93 | (0.73, 1.17) | 0.543 |

*OR: odds ratio for every 10% increase in European ancestry.
