# Supplementary material for: African genetic ancestry interacts with body mass index to modify risk for uterine fibroids
Source: PLoS Genet. 2017 Jul 17;13(7):e1006871. doi: 10.1371/journal.pgen.1006871 (PMC5536439; doi:10.1371/journal.pgen.1006871)
Supplement: S4 Table — (DOCX) [file pgen.1006871.s004.docx]

**S4 Table: Detailed estimates from top two regions from local ancestry x BMI (continuous) interaction models represented in Table 3 and Table 5 in the main document.**

|  |  | **BioVU** | |  | **CARDIA** | |
| --- | --- | --- | --- | --- | --- | --- |
| **Region** | **Variable** | **OR** | **P** |  | **OR** | **P** |
| **chr 6p24 Local Ancestry at rs6457825** | Local ancestry | 3.8 | 0.002 |  | 2.58 | 0.2558 |
|  | BMI | 1.036 | 1.80E-05 |  | 1.04 | 0.018 |
|  | Local anc x BMI | 0.95 | 0.000236 |  | 0.95 | 0.062 |
|  |  |  |  |  |  |  |
| **chr 2q31-32 Local ancestry at rs12999125** | Local ancestry | 0.2252 | 0.0008143 |  | 0.222 | 0.08651 |
|  | BMI | 1.005 | 0.5609 |  | 1.004 | 0.8116 |
|  | Local anc x BMI | 1.043 | 0.0013 |  | 1.052 | 0.06264 |

*Models were run with BMI as a continuous variable, local ancestry coded as 0, 1, 2, and a continuous interaction term of local ancestry and BMI.
