# Supplementary material for: African genetic ancestry interacts with body mass index to modify risk for uterine fibroids
Source: PLoS Genet. 2017 Jul 17;13(7):e1006871. doi: 10.1371/journal.pgen.1006871 (PMC5536439; doi:10.1371/journal.pgen.1006871)
Supplement: S5 Table — (DOCX) [file pgen.1006871.s005.docx]

**S5 Table. Top 10 SNPs from Meta-analysis of BMI x SNP interaction estimates from two candidate genetic-regions in BioVU AA, CARDIA AA and BioVU EA.**

|  |  |  |  | **BIOVU AA** | |  | **CARDIA AA** | |  | **Meta-analysis AA** | | |  | **BIOVU EA (Replication)** | |  | **Meta-analysis AA + EA** | | |
| --- | --- | --- | --- | --- | --- | --- | --- | --- | --- | --- | --- | --- | --- | --- | --- | --- | --- | --- | --- |
| **SNP** | **CHR** | **BP** | **A1/A2** | **BETA** | **P** |  | **BETA** | **P** |  | **BETA** | **P** | **I^2^** |  | **BETA** | **P** |  | **BETA** | **P** | **I^2^** |
| rs303051 | 6 | 10404196 | G/A | 0.038 | 2.27E-04 |  | 0.049 | 1.27E-02 |  | 0.041 | 9.65E-06 | 0 |  | 0.017 | 1.59E-01 |  | 0.032 | 1.34E-05 | 30 |
| rs111396056 | 6 | 10402438 | CAG/C | 0.038 | 2.52E-04 |  | 0.048 | 1.38E-02 |  | 0.040 | 1.14E-05 | 0 |  | 0.014 | 2.39E-01 |  | 0.030 | 2.83E-05 | 40 |
| rs10948377* | 6 | 12987237 | A/G | 0.064 | 2.26E-03 |  | 0.043 | 2.53E-01 |  | 0.059 | 1.26E-03 | 0 |  | 0.029 | 3.65E-03 |  | 0.036 | 4.21E-05 | 14 |
| rs55844045 | 6 | 10552533 | T/TA | 0.043 | 7.85E-05 |  | 0.045 | 9.35E-02 |  | 0.043 | 1.79E-05 | 0 |  | 0.013 | 3.34E-01 |  | 0.032 | 6.16E-05 | 39 |
| rs4711899* | 6 | 12986466 | T/C | 0.018 | 7.07E-02 |  | 0.075 | 7.78E-04 |  | 0.027 | 2.52E-03 | 82 |  | 0.022 | 1.14E-02 |  | 0.025 | 8.76E-05 | 64 |
| rs303048 | 6 | 10398982 | T/A | 0.037 | 4.21E-04 |  | 0.047 | 1.84E-02 |  | 0.039 | 2.46E-05 | 0 |  | 0.011 | 3.71E-01 |  | 0.028 | 1.05E-04 | 47 |
| rs303049 | 6 | 10400157 | G/C | 0.031 | 2.33E-03 |  | 0.055 | 1.43E-02 |  | 0.035 | 1.53E-04 | 0 |  | 0.017 | 1.89E-01 |  | 0.029 | 1.20E-04 | 9 |
| rs503532* | 6 | 10389971 | T/C | 0.034 | 2.21E-03 |  | 0.022 | 3.21E-01 |  | 0.032 | 1.46E-03 | 0 |  | 0.022 | 2.45E-02 |  | 0.027 | 1.30E-04 | 0 |
| rs7750120* | 6 | 13072633 | C/T | 0.033 | 1.31E-03 |  | 0.015 | 4.63E-01 |  | 0.030 | 1.36E-03 | 0 |  | 0.022 | 3.03E-02 |  | 0.026 | 1.32E-04 | 0 |
| rs9463423* | 6 | 13069359 | C/T | 0.033 | 1.30E-03 |  | 0.015 | 4.79E-01 |  | 0.030 | 1.40E-03 | 0 |  | 0.022 | 3.03E-02 |  | 0.026 | 1.35E-04 | 0 |
|  |  |  |  |  |  |  |  |  |  |  |  |  |  |  |  |  |  |  |  |
| rs1429421* | 2 | 185915220 | A/T | 0.040 | 8.73E-03 |  | 0.075 | 3.95E-02 |  | 0.046 | 1.29E-03 | 0 |  | 0.026 | 3.94E-03 |  | 0.031 | 3.33E-05 | 9 |
| rs5836927* | 2 | 185762137 | AT/A | 0.038 | 1.26E-02 |  | 0.056 | 1.17E-01 |  | 0.040 | 3.65E-03 | 0 |  | 0.025 | 3.79E-03 |  | 0.030 | 6.29E-05 | 0 |
| rs71430182* | 2 | 186190060 | G/A | 0.023 | 3.44E-02 |  | 0.040 | 4.98E-02 |  | 0.027 | 5.36E-03 | 0 |  | 0.036 | 3.74E-03 |  | 0.030 | 7.15E-05 | 0 |
| rs1344706* | 2 | 185778428 | C/A | 0.032 | 6.55E-02 |  | 0.079 | 7.63E-02 |  | 0.038 | 1.82E-02 | 0 |  | 0.028 | 1.70E-03 |  | 0.031 | 1.00E-04 | 0 |
| rs17510170* | 2 | 185876645 | T/A | 0.036 | 1.44E-02 |  | 0.056 | 9.91E-02 |  | 0.039 | 3.70E-03 | 0 |  | 0.024 | 6.15E-03 |  | 0.029 | 1.05E-04 | 0 |
| rs13388087* | 2 | 185734704 | C/G | 0.032 | 2.98E-02 |  | 0.056 | 1.13E-01 |  | 0.035 | 8.96E-03 | 0 |  | 0.026 | 3.42E-03 |  | 0.029 | 1.05E-04 | 0 |
| rs13401381* | 2 | 185734749 | C/T | 0.032 | 2.98E-02 |  | 0.056 | 1.13E-01 |  | 0.035 | 8.96E-03 | 0 |  | 0.026 | 3.42E-03 |  | 0.029 | 1.05E-04 | 0 |
| rs10210216* | 2 | 185740015 | G/A | 0.031 | 3.41E-02 |  | 0.056 | 1.14E-01 |  | 0.035 | 1.04E-02 | 0 |  | 0.026 | 3.33E-03 |  | 0.028 | 1.14E-04 | 0 |
| rs11681373* | 2 | 185785791 | G/A | 0.032 | 6.85E-02 |  | 0.081 | 7.09E-02 |  | 0.038 | 1.87E-02 | 5 |  | 0.028 | 1.90E-03 |  | 0.030 | 1.15E-04 | 0 |
| rs7583540* | 2 | 185834395 | T/C | 0.035 | 1.69E-02 |  | 0.057 | 9.56E-02 |  | 0.039 | 4.30E-03 | 0 |  | 0.024 | 6.29E-03 |  | 0.028 | 1.21E-04 | 0 |

*Represents candidate SNPs from BioVU AA and CARDIA AA meta-analysis which also replicated (p < 0.05) in BioVU EA.
