# Supplementary material for: African genetic ancestry interacts with body mass index to modify risk for uterine fibroids
Source: PLoS Genet. 2017 Jul 17;13(7):e1006871. doi: 10.1371/journal.pgen.1006871 (PMC5536439; doi:10.1371/journal.pgen.1006871)
Supplement: S6 Table — (DOCX) [file pgen.1006871.s006.docx]

**S6 Table: Association between BMI and fibroid presence by race/ethnicity status in CARDIA**

| Variable | Whites (Total N = 497) | | |  | Blacks (Total N = 463) | | | P-int** |
| --- | --- | --- | --- | --- | --- | --- | --- | --- |
|  | OR | 95% CI | P |  | OR | 95% CI | P* |  |
| **BMI** |  |  |  |  |  |  |  | 0.881 |
| <25kg/m2 | 1.00 | (Ref) |  |  | 1.00 | (Ref) |  |  |
| 25-30 kg/m2 | 1.65 | (1.06, 2.57) | 0.026 |  | 1.58 | (0.85, 2.95) | 0.151 |  |
| 30-35 kg/m2 | 1.24 | (0.68, 2.25) | 0.472 |  | 1.17 | (0.65, 2.11) | 0.610 |  |
| >35 kg/m2 | 1.53 | (0.87, 2.69) | 0.139 |  | 1.18 | (0.66, 2.10) | 0.577 |  |
| Models Adjusted for cws_age and diabetes status | | | | | | | | |
| *P: P-value from Wald-test for individual categories | | | | | | | | |
| **P-int: P-value for global interaction using likelihood ratio test | | | | | | | | |
