# Supplementary material for: African genetic ancestry interacts with body mass index to modify risk for uterine fibroids
Source: PLoS Genet. 2017 Jul 17;13(7):e1006871. doi: 10.1371/journal.pgen.1006871 (PMC5536439; doi:10.1371/journal.pgen.1006871)
Supplement: S7 Table — (DOCX) [file pgen.1006871.s007.docx]

**S7 Table: Sensitivity analyses comparing race and BMI interactions for CARDIA: Using CWS ultrasound confirmed cases and controls only vs. using ultrasound confirmed cases and controls and self-reported hysterectomy due to fibroids as additional cases.**

|  | **CWS Ultrasound Confirmed and Fibroids-as Cause for Hysterectomy Combined** | | | | |  | **CWS Ultrasound Confirmed Only** | | | | |
| --- | --- | --- | --- | --- | --- | --- | --- | --- | --- | --- | --- |
| BMI Categories | N Cases/Controls | OR^a^ | (95% CI) | P^b^ | P-int^c^ |  | N Cases/Controls | OR^a^ | (95% CI) | P^b^ | P-int^c^ |
| <25kg/m2 | 129/184 | 0.22 | (0.12, 0.39) | <0.001 | 0.88 |  | 144/182 | 0.18 | (0.10, 0.32) | <0.001 | 0.75 |
| 25-30 kg/m2 | 142/103 | 0.31 | (0.18, 0.55) | <0.001 |  |  | 179/99 | 0.25 | (0.14, 0.43) | <0.001 |  |
| 30-35 kg/m2 | 109/80 | 0.36 | (0.18, 0.74) | 0.005 |  |  | 128/80 | 0.36 | (0.18, 0.71) | 0.003 |  |
| >35 kg/m2 | 128/90 | 0.42 | (0.22, 0.79) | 0.007 |  |  | 156/85 | 0.35 | (0.19, 0.66) | 0.001 |  |

^a^OR: whites (comparison group), blacks (reference group)
^b^P: P-value from z-score for individual categories
^c^P-int: P-value for global interaction using likelihood ratio test
