# Supplementary material for: African genetic ancestry interacts with body mass index to modify risk for uterine fibroids
Source: PLoS Genet. 2017 Jul 17;13(7):e1006871. doi: 10.1371/journal.pgen.1006871 (PMC5536439; doi:10.1371/journal.pgen.1006871)
Supplement: S1 Fig — (PDF) [file pgen.1006871.s008.pdf]

S1 Fig. Visual representation of the interaction between reported race and BMI-categories in the Synthetic Derivative

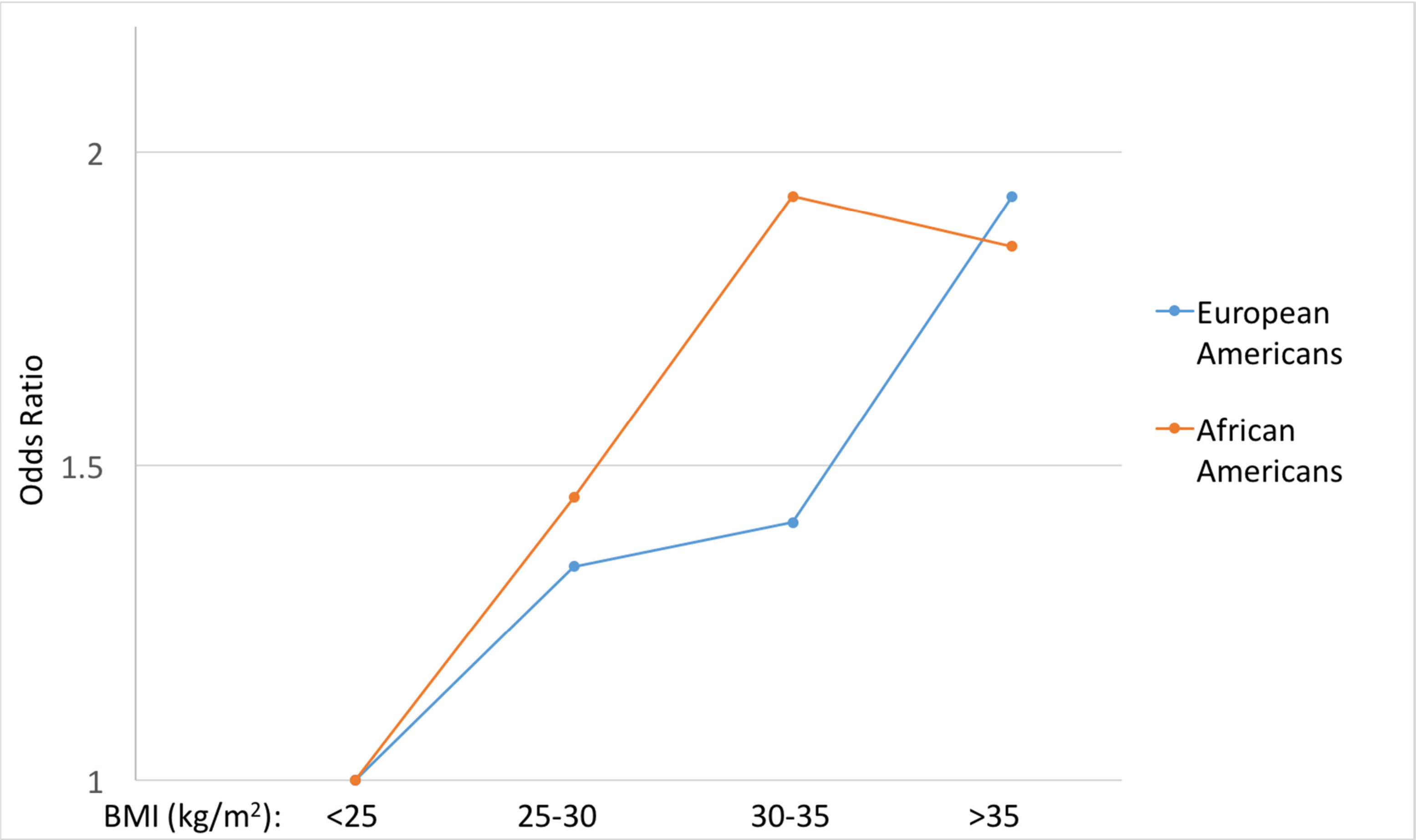

Effect estimates, confidence intervals and corresponding p-values for associations visualized here are shown in S1 Table
