# Supplementary material for: African genetic ancestry interacts with body mass index to modify risk for uterine fibroids
Source: PLoS Genet. 2017 Jul 17;13(7):e1006871. doi: 10.1371/journal.pgen.1006871 (PMC5536439; doi:10.1371/journal.pgen.1006871)
Supplement: S2 Fig — Negative log(10) p-values for fibroids modeled against local European ancestry and BMI with continuous interaction term (BMI x Local ancestry). (PDF) [file pgen.1006871.s009.pdf]

S2 Fig. Strongest ancestry by BMI interactions in meta-analysis: Chromosome 6

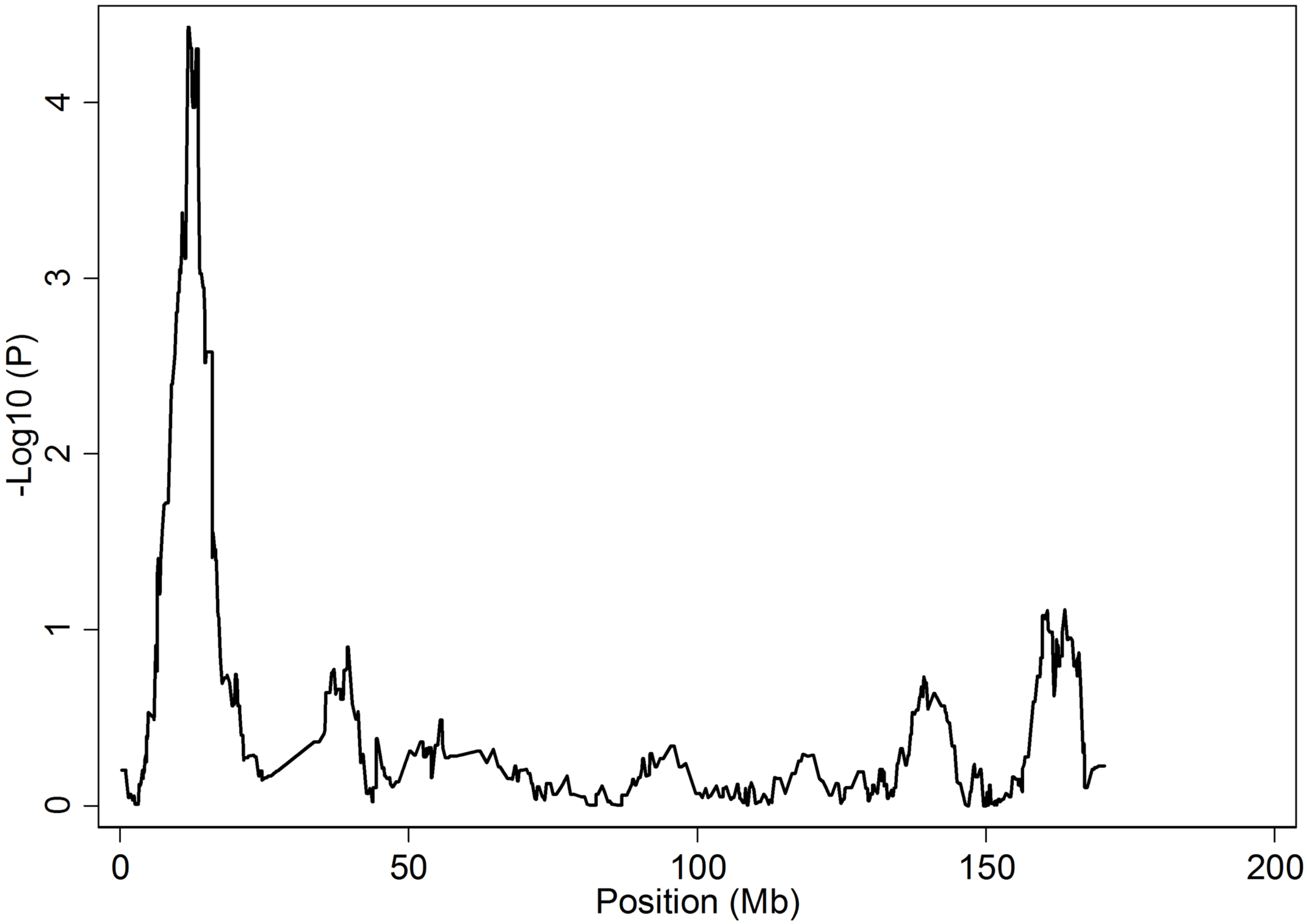

Negative log(10) P values for fibroids modeled against local European ancestry and BMI with continuous interaction term (BMI x Local ancestry)
