# Supplementary material for: African genetic ancestry interacts with body mass index to modify risk for uterine fibroids
Source: PLoS Genet. 2017 Jul 17;13(7):e1006871. doi: 10.1371/journal.pgen.1006871 (PMC5536439; doi:10.1371/journal.pgen.1006871)
Supplement: S3 Fig — (PDF) [file pgen.1006871.s010.pdf]

S3 Fig. Regional association plots for SNPs under admixture mapping peak with the strongest overall and stratified by BMI categories

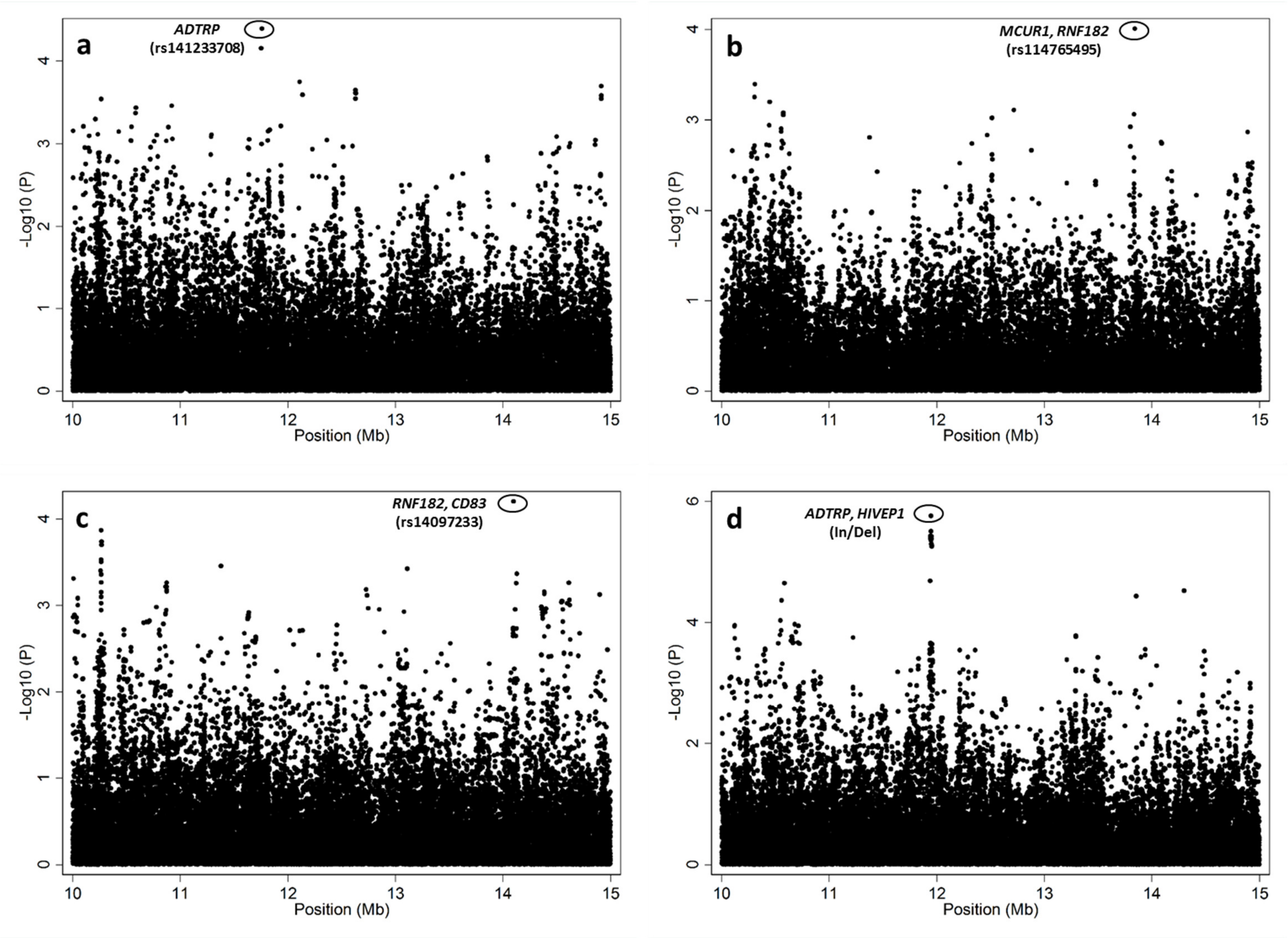

a = Overall; b=Normal weight; c=Overweight; d=Obese
