# Supplementary figures and images for: African genetic ancestry interacts with body mass index to modify risk for uterine fibroids
Source: PLoS Genet. 2017 Jul 17;13(7):e1006871. doi: 10.1371/journal.pgen.1006871 (PMC5536439; doi:10.1371/journal.pgen.1006871)

**S4 Fig. Association plot for local ancestry x BMI (continuous) interaction in chromosome 2**

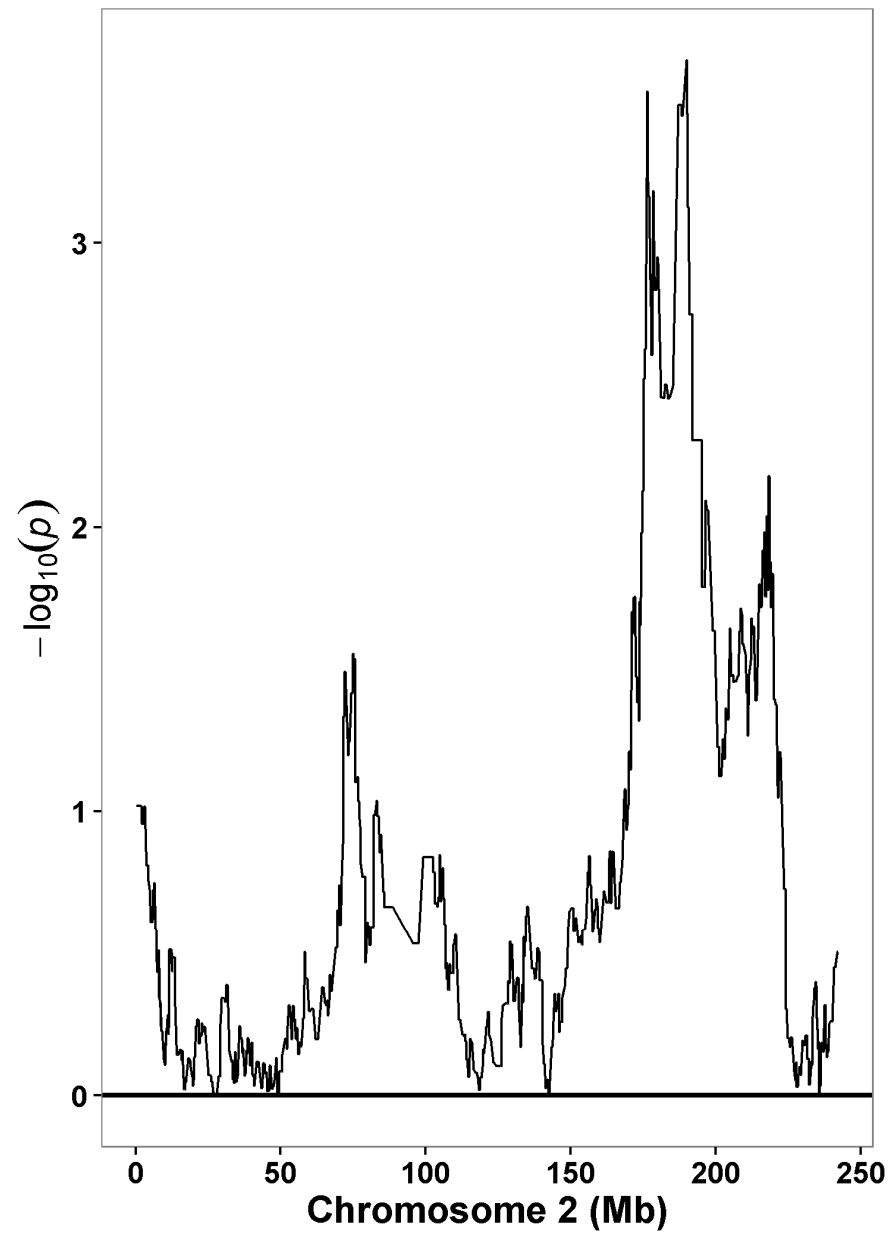

Supplement: S4 Fig — (PDF) [file pgen.1006871.s011.pdf]
