# Supplementary material for: African genetic ancestry interacts with body mass index to modify risk for uterine fibroids
Source: PLoS Genet. 2017 Jul 17;13(7):e1006871. doi: 10.1371/journal.pgen.1006871 (PMC5536439; doi:10.1371/journal.pgen.1006871)
Supplement: S6 Fig — (PDF) [file pgen.1006871.s013.pdf]

S6 Fig. Comparing proximity of markers from previously published admixture mapping signals for chromosome 2q31-q33.

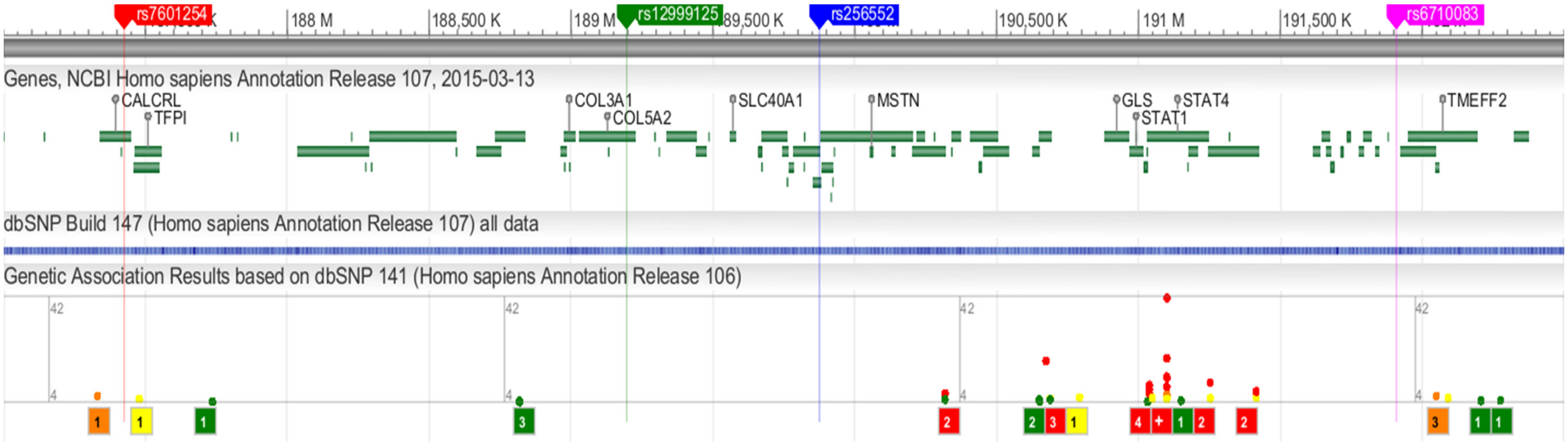

Admixture mapping markers from present study: Red SNP tag (rs7601254) and Green SNP tag (rs129999125); Admixture mapping markers from Zhang et al.: Blue SNP tag (rs256552); Admixture mapping marker from Wise et al.: Pink SNP tag (rs6710083)
