# Supplementary material for: African genetic ancestry interacts with body mass index to modify risk for uterine fibroids
Source: PLoS Genet. 2017 Jul 17;13(7):e1006871. doi: 10.1371/journal.pgen.1006871 (PMC5536439; doi:10.1371/journal.pgen.1006871)

S7 Fig. Comparison of average European ancestry estimates from LAMP local ancestry calls with the first principal component in African American women from a) BioVU and b) CARDIA

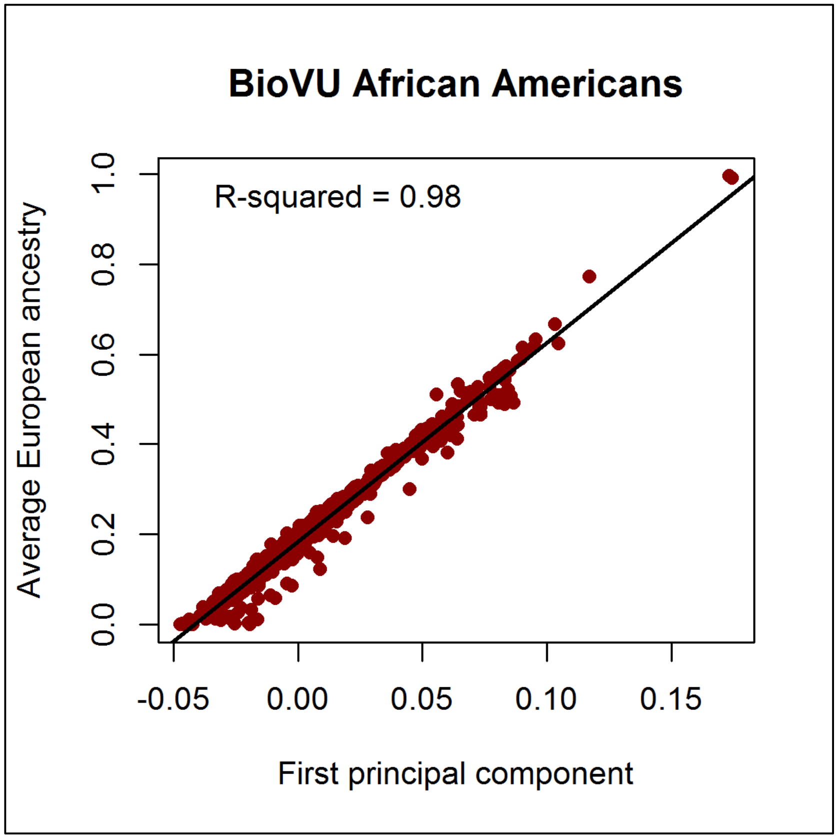

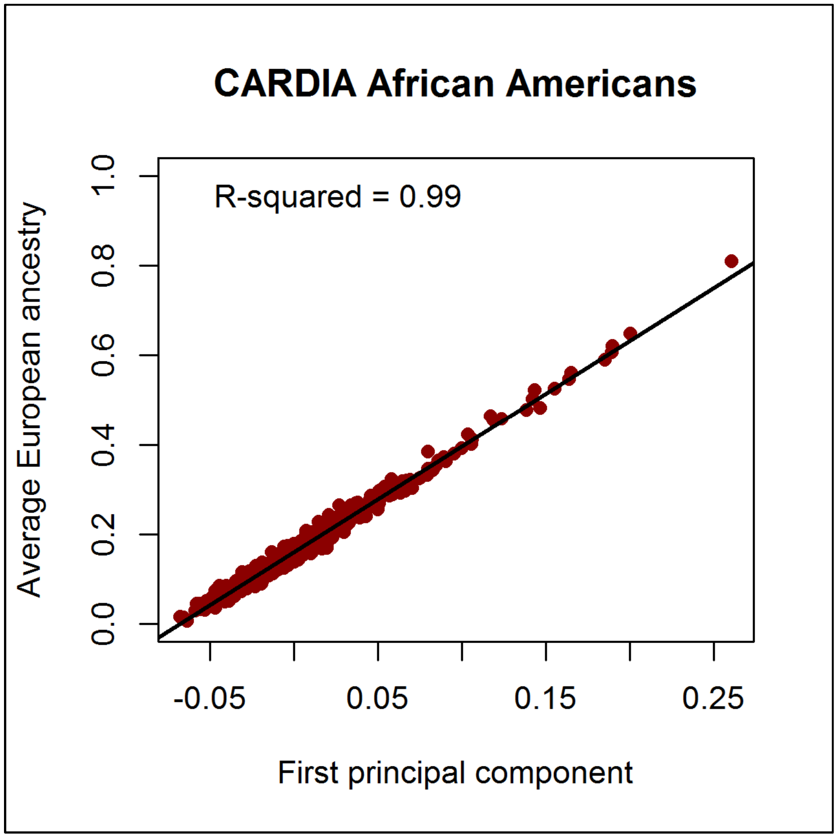

Supplement: S7 Fig — (PDF) [file pgen.1006871.s014.pdf]
